# Childhood cancer research in Oxford II: The Childhood Cancer Research Group

**DOI:** 10.1038/s41416-018-0181-z

**Published:** 2018-08-21

**Authors:** Gerald J. Draper, John F. Bithell, Kathryn J. Bunch, Gerald M. Kendall, Michael F. G. Murphy, Charles A. Stiller

**Affiliations:** 1 Department of Statistics, University of Oxford, 24-29 St Giles, Oxford, OX1 3LB UK; 2 National Perinatal Epidemiology Unit, Nuffield Department of Population Health, University of Oxford, Richard Doll Building, Old Road Campus, Oxford, OX3 7LF UK; 3 Cancer Epidemiology Unit, Nuffield Department of Population Health, University of Oxford, Richard Doll Building, Old Road Campus, Oxford, OX3 7LF UK; 4 Nuffield Department of Women’s and Reproductive Health, University of Oxford, John Radcliffe Hospital, Oxford, OX3 9DU UK; 5 National Cancer Registration and Analysis Service, Public Health England, Chancellor Court, Oxford Business Park South, Oxford, OX4 2GX UK

**Keywords:** Oncology, Risk factors

## Abstract

**Background:**

We summarise the work of the Childhood Cancer Research Group, particularly in relation to the UK National Registry of Childhood Tumours (NRCT).

**Methods:**

The Group was responsible for setting up and maintaining the NRCT. This registry was based on notifications from regional cancer registries, specialist children’s tumour registries, paediatric oncologists and clinical trials organisers. For a large sample of cases, data on controls matched by date and place of birth were also collected.

**Results:**

Significant achievements of the Group include: studies of aetiology and of genetic epidemiology; proposals for, and participation in, international comparative studies of these diseases and on a classification system specifically for childhood cancer; the initial development of, and major contributions to, follow-up studies of the health of long-term survivors; the enhancement of cancer registration records by the addition of clinical data and of birth records. The Group made substantial contributions to the UK government’s Committee on Medical Aspects of Radiation in the Environment.

**Conclusion:**

An important part of the ethos of the Group was to work in collaboration with many other organisations and individuals, both nationally and internationally: many of the Group’s achievements described here were the result of such collaborations.

## Introduction

This paper is one of three summarising research on childhood cancer done mainly in Oxford over six decades starting in 1954. The main intention is to summarise the history and achievements of this research. It is our hope that these papers will also serve to draw attention to the availability of the very substantial research resources accumulated over this period. The first paper^1^ describes the Oxford Survey of Childhood Cancers (OSCC) which was initiated by Dr Alice Stewart at the University of Oxford Department of Social Medicine. At the time of Alice Stewart’s retirement in 1974 the Standing Subcommittee on Cancer of the DHSS Standing Medical Advisory Committee recommended the setting up of a national registry of childhood malignancies. The Childhood Cancer Research Group (CCRG) was established in 1975 within the department of Sir Richard Doll, the Regius Professor of Medicine. The first director was GJ Draper, and he was succeeded in 2002 by MFG Murphy. The remit of the CCRG included maintaining what became the National Registry of Childhood Tumours (NRCT) and conducting research into the epidemiology of these diseases; from the outset it was assumed that the group would be involved in collaborative studies.

The present paper is one of two describing the genesis and achievements of the work done by the CCRG. It is confined to research studies based on, or including, data from the NRCT or the OSCC. A large part of the work of the Group was concerned with studies investigating carcinogenic effects of ionising radiation; this is dealt with in the accompanying paper by Kendall et al.^2^

Other CCRG work on childhood cancer is included in the list of references on the journal website (http://www.nature.com/bjc). This includes a series of papers resulting from participation in the Inter-regional Epidemiological Study of Childhood Cancer, a case–control study involving cases from three regions of England.

In this paper, we have briefly summarised some of the main findings and conclusions from the epidemiological and other studies based on OSCC/CCRG/NRCT data. But no attempt has been made to summarise the findings of the very large number of clinical/pathological studies that the CCRG facilitated or the international studies of incidence and survival for which the CCRG/NRCT was often a major contributor.

## Materials and methods

The records on which the NRCT was initially based were those collected for the OSCC by Alice Stewart and her colleagues. These included records relating to children diagnosed with malignant neoplasms, and non-malignant intracranial and intraspinal tumours, in England, Scotland and Wales from 1962 onwards, together with records of deaths of children treated in earlier years that had initially been collected as part of Stewart’s work. From 1993, the coverage was extended to Northern Ireland. The most important characteristic of the NRCT was the extent to which the dataset routinely collected by cancer registries in the UK was expanded to include notifications from the national group of paediatric oncologists (the Children’s Cancer and Leukaemia Group, CCLG), birth registration records, information on congenital anomalies and genetic conditions, detailed pathology records and information from clinical trials. In addition, for a large sub-group of the cases, a control series was established, with birth registration records, initially for one control per case, matched by sex and by date and area of birth and, for England and Wales, provided by the Office for National Statistics (ONS). Similar controls were also obtained for Scotland. The resulting database became a resource for geographical and other case–control studies (e.g. on exposure to electric and magnetic fields (EMF), gamma radiation and radon). To improve the power of later studies two matched controls per case were selected for cases born from 2000 onwards. By 2014, when the CCRG closed, information had been collected for about 57,000 cases born and registered in the UK, 1962–2010, and for about 73,000 controls.

Much of the work of the Group was concerned with epidemiological studies and with studies of the natural history and pathology of particular tumours. A major component of the early epidemiological work consisted in studying the possible carcinogenic effects of ionising radiation. Initially, this was a response to a television programme broadcast in 1983—“Windscale, the Nuclear Laundry” that identified a larger than expected number of cases of childhood leukaemia in the vicinity of this nuclear installation, subsequently renamed and better known as Sellafield. The excess was sufficient to cause alarm and to trigger a major enquiry^3^ which confirmed that the incidence in the nearby village of Seascale was well beyond normal expectation. This enquiry led to the establishment of a Government advisory committee—the Committee on Medical Aspects of Radiation in the Environment (COMARE). A discussion of the work of the CCRG in relation to this committee is included in the accompanying paper by Kendall et al.^2^

The CCRG worked with many other groups and individuals, both nationally and internationally. Two very long-term collaborations were especially important. The first was with other cancer registration organisations in the UK, both nationally—the ONS, previously the Office for Population Censuses and Surveys (OPCS)—and regionally, particularly the regional Childhood Tumour Registries; the NRCT was a member of the UK Association of Cancer Registries and latterly of the National Cancer Intelligence Network. The second major collaboration was with the CCLG (formerly UK Children’s Cancer Study Group, UKCCSG) and with individual paediatric oncologists and other clinicians.

The inclusion of birth records and in particular parental information in the NRCT made it possible, using record linkage techniques, and in collaboration with organisations holding other datasets, for the CCRG to investigate relationships between childhood cancer and putative aetiological factors. Studies of parental age and sibship position and of assisted reproductive technology are described below; studies of parents potentially exposed to ionising radiation are described in Kendall et al.^2^ Record linkage was also used to identify childhood cancer survivors who later underwent cardiac transplantation as described below.

## Results

### UK studies of incidence, survival and follow-up

The CCRG carried out a series of analyses of childhood cancer incidence and survival based on the NRCT, and using standard classification systems appropriate to childhood tumours. The most comprehensive national data on incidence, survival and mortality were published in the OPCS volume by Draper et al.^4^ and the monograph edited by Stiller.^5^ Detailed incidence data were also published in a paper describing the methodology of the NRCT.^6^

#### Time trends

Time trends in incidence were analysed in Draper et al.^7^ and Stiller.^5^ In the latter it was concluded that in Britain there was a total increase in recorded incidence of 38% over the 35-year period 1966 to 2000. The increase was seen, in varying degrees over all the main diagnostic groups of childhood cancers; the authors commented that “These increases in recorded incidence do not necessarily represent real changes in risk …” but suggested that the change was real at least for acute lymphoblastic leukaemia (ALL) and malignant melanoma. In a study that documented increasing time trends in recorded incidence of subtypes of childhood leukaemia, Kroll et al.^8^ noted that two separate influenza epidemics each coincided with a small peak in childhood ALL. Kroll et al.^9^ validated the completeness of NRCT ascertainment for cases of childhood cancer diagnosed during 2003–04. Kroll et al.^10^ suggested that improved completeness of diagnosis and registration “were plausible explanations for most of the changes in recorded incidence” but that “the possibility of some real increases should not be ruled out”.

#### Survival analyses

The 1982 report included the first results on national population-based survival for all the principal diagnostic groups, covering children diagnosed to the end of 1974.^4^ This was followed by detailed studies of survival trends among children diagnosed during 1971–85^11^ and 1980–91,^12^ the latter including projected long-term survival estimates for recently diagnosed patients using a rudimentary form of the period approach *avant la lettre* cf.^13^ The 2007 monograph included survival analyses for all children diagnosed during 1966–2000.^5^ These studies documented the remarkable increase in survival rates from childhood cancer between the 1960s, when five-year survival was below 30%, and 30 years later, when it exceeded 75%. Stiller et al.^14^ demonstrated how changes in population-based survival for a wide range of childhood cancers paralleled those reported from the relevant clinical trials during 1978 onwards.

Shah et al.^15^ applied new methods to define the proportion of children with leukaemia who appeared to be cured—i.e. who, as a group, eventually experience no excess mortality compared with the general population. This rose from 25% for those diagnosed in 1971–1975 to 68% in 1991–1995, though the average time since diagnosis at which cure could reasonably be declared also increased, from 11 years for those diagnosed during 1971–1975 to 16 years for those diagnosed during 1986–1990, perhaps because of late relapse, secondary malignancy and toxicity from treatment.^16^ Stiller et al.^14^ demonstrated how changes in population-based survival for a wide range of childhood cancers paralleled those reported from the relevant clinical trials

International collaborative studies of incidence and survival are described below.

### The health of survivors

From the foundation of the CCRG the long-term health of survivors was recognised as a major object of concern, and a research programme was started. Hawkins et al.^17^ in a follow-up study of 10,106 three-year survivors, confirmed the previously reported high risk of second primary cancers among patients with heritable retinoblastoma and found that for all other diagnostic groups combined the number of subsequent cancers was five times the population rate. Hawkins et al.^18,19^ studied the offspring of survivors of childhood malignant disease and, albeit on the basis of fairly small numbers and short follow-up, found no evidence of mutagenic effects of therapy on the offspring and only previously recognised patterns of inheritance. Much of the subsequent work on the health of survivors has been carried out by MM Hawkins in the British Childhood Cancer Survivor Study (BCCSS), at the University of Birmingham. The BCCSS cohort was derived from the NRCT and some of the work was done in collaboration with the CCRG.

The very high risk of other forms of cancer in carriers of the retinoblastoma gene is of particular concern; CCRG studies are included in the section on retinoblastoma below.

Another particular area of concern is the risk among childhood cancer survivors of adverse cardiac effects following treatment. Two studies^20,21^ quantified the need for cardiac transplantation among those affected, and assessed their survival post-transplantation. Taken together, these studies demonstrate an increasing need for transplantation among childhood cancer survivors. This is most marked for survivors of acute myeloid leukaemia, whose increased survival rates have been achieved as a result of high doses of cardiotoxic anthracyclines.

### Studies of familial factors in childhood cancer

#### Sibs

The NRCT, having inherited data from the OSCC,^1^ and continued to ascertain cases for a further 40 years, included one of the largest sets of data in the world on childhood cancer in sibs. Papers on familial risks and patterns of occurrence were published.^22,23^ In the first, as reported in,^1^ estimates were made of the cancer risks to sibs of cases. It was concluded that, when cases with certain obviously genetic conditions were excluded, the risk that a sib of a child with cancer would also be affected by cancer below age 15 years was double the normal risk. In the absence of known environmental factors that could account for this risk, it seems reasonable, for genetic counselling purposes, to act on the assumption that this increase is a consequence of a shared genetic background.

Winther et al.^24^ using data from the Nordic countries, concluded that given information on family histories and the ability to recognise syndromes such as Li-Fraumeni, there was no unexplained increase in the risk to sibs of cases. This does not, however contradict the finding of Draper et al.;^23^ the point here is that, although the finding of an increased risk can be explained by information obtained *ex post facto*, the genetic counselling situation is that the question of whether a sib of a case has an increased risk will often have to be assessed in the absence of such information. Draper et al.^23^ calculated the risk to a future sib on the basis of the information that the proband had cancer but in the absence of prior knowledge about genetic disease in the family; this is the relevant risk for genetic counselling purposes. Winther et al.^24^ calculated the risk for sibs excluding families where there is evidence (including that from the second sib) that there is a known familial syndrome; this is relevant to the question of whether, after allowing for these known syndromes, there are still unrecognised genetic factors associated with childhood cancer. (A second difference between these studies is that the former findings relate to the age-group 0–14 years; the latter to age-group 0–19 years.)

#### Twins

If one member of a twin pair is affected by childhood cancer there seems to be an increased risk that the co-twin will be affected by the same disease.^22,25^ The rarity of childhood cancers and of monozygotic twins means that it has been impossible to give estimates of concordance rates except for leukaemia. For this disease Buckley et al.^25^ estimated that the concordance rate for monozygotic twins is 5%. Chaganti et al.^26^ and Ford et al.^27^ have shown that this concordance can, in at least some cases, be explained by intraplacental transfer of transformed cells.

Twins are less likely than singletons to develop childhood malignant disease. Hewitt et al.^28^ suggested that this was because a member of a pair affected in utero may have an increased risk of dying before the twin pregnancy is recognised as such. See also Bithell et al.^1^ and Murphy et al.^29,30^ suggested other possible explanations, e.g. the lower-than-average birthweight of twins.

#### Retinoblastoma

Retinoblastoma is of particular interest and concern, mainly because of the large proportion (40–45%) of cases that have a well-understood genetic origin, and the fact that RB1 gene mutations are associated with other cancers in addition to retinoblastoma. CCRG studies included analyses of survival and the incidence among survivors of second primary tumours at other sites.^31–33^ For some decades, it has been recognised that survivors are at risk of bone and soft-tissue cancers. But as follow-up periods increased it became clear that this risk extends to a wide variety of other types of cancer. A study of cancer rates among relatives of cases^34^ was among the first to document the high continuing lifetime risk of other cancers among carriers of retinoblastoma gene mutations.

Draper et al.^35^ gave estimates of the risk of retinoblastoma for sibs and offspring of cases according to whether or not these probands had the heritable form of the disease and whether it was unilateral or bilateral.

#### Other genetic conditions and congenital anomalies

Recording of congenital anomalies and genetic conditions in the NRCT made possible studies of the heritable fraction of childhood cancer^36^ and of childhood cancer risk associated with a wide range of anomalies^37^ and also investigations related to specific syndromes. The association between hepatoblastoma and polyposis coli was first reported by Kingston e al.^38^ who studied 113 cases and estimated on the basis of this, admittedly small, sample that mothers of children with hepatoblastoma had about 200 times the population risk of polyposis coli—or, if it is assumed that the association applies equally to fathers, that the risk of a parent being affected is 100 times the population risk. Three studies^39–41^ reported on survival of children with Down syndrome and leukaemia, and the NRCT made a major contribution to international studies highlighting the apparent protective effect of trisomy 21 against neuroblastoma and medulloblastoma.^42,43^ Population-based estimates of the risk of leukaemia and lymphoma associated with neurofibromatosis were calculated by Stiller et al.,^44^ several years before it began to be recognised that a large proportion of patients apparently affected with this rare combination of conditions had what has come to be known as constitutional mismatch-repair deficiency syndrome. The CCRG was a long-term member of the Factors Associated with Childhood Tumours (FACT) collaboration, based at the Institute of Cancer Research, whose series of studies has identified several genes involved in the development of embryonal and other tumours.^45–52^

### Aetiological studies

#### Ionising radiation

The CCRG carried out a series of studies on the possible effects of ionising radiation on the incidence of childhood cancer; these are described in detail in the associated paper by Kendall et al.^2^ Much of this work was done in collaboration with the National Radiological Protection Board, which later became part of the Health Protection Agency and then Public Health England.

#### Non-ionising radiation

Possible effects of electric and magnetic fields near high-voltage powerlines have been studied in collaboration with John Swanson of the National Grid Company. An early study^53^ reported an increased leukaemia risk for children with a residential address at birth within 600 m of a high-voltage powerline. Subsequent analyses demonstrated that this leukaemia risk declined over time from a significant increase in the 1960s and 1970s to no increase or a non-significant decrease in the more recent decades.^54^ These unexpected findings relating to distance from powerlines are not consistent with the existing body of evidence on magnetic fields^55,56^ and have prompted replications in other countries^57–59^ that did not show similar distance effects. Further CCRG studies found no significant association between cancer risk and proximity to high-voltage underground cables^55^ nor could the observed effects of overhead lines be explained by the corona-ion hypothesis.^60^ It was, however, concluded that some link between the presence of high-voltage powerlines and socioeconomic or demographic factors in the vicinity was the most likely explanation.^61^

NRCT data on powerlines have been contributed to three international pooled studies of childhood cancer. Two examined magnetic fields, and confirmed the previously reported association for leukaemia but provided little evidence of an association for brain tumours). The third,^62^ prompted directly by the previous UK findings,^53,54^ examined residential distance from high-voltage powerlines in relation to childhood leukaemia risk. This new pooled analysis found no material association between childhood leukaemia and distance to the nearest overhead powerline of any voltage, but a small though non-significantly increased risk for children living <50 m from 200+ KV powerlines. The distinctive features of the previous UK findings were not confirmed in other countries and no clearer explanation for the increased risks with distance found in various studies has been revealed.

#### Vitamin K

Golding et al.^63,64^ reported a possible doubling of the risk of childhood cancer from intramuscular vitamin K, given to babies to prevent vitamin K deficiency bleeding. Two CCRG studies^65,66^ were carried out to investigate this suggestion; these authors concluded that there might be a small effect on the incidence of childhood leukaemia but none for other cancers. Roman et al.^67^ in a pooled analysis of six case–control studies, including the CCRG study, concluded that while “…small effects cannot be entirely ruled out, our analysis provides no convincing evidence that intramuscular vitamin K is associated with childhood leukaemia”. And there was even less evidence of a risk for the combined group of other childhood cancers.

#### Assisted reproductive technology

There has long been wide interest^68^ in the possibility of there being a differential childhood cancer risk in children born following assisted reproduction and in particular in vitro fertilisation (IVF). The NRCT with its complete coverage of childhood tumours in Great Britain offered a unique resource for addressing this question. Data on children born after IVF treatment between 1978 and 1991 were initially held by the Medical Research Council, and record linkage techniques were used to establish the numbers of such children who went on to develop childhood cancer.^69^ The Human Fertilisation and Embryology Authority (HFEA) took over responsibility for record keeping for assisted births from 1992 onwards. Although the HFEA was committed to monitoring the long-term outcomes for children born following assisted reproduction interventions, legislation had precluded any release of data on individuals. A major revision of the Human Fertilisation and Embryology Act in 2008 allowed approved researchers highly regulated access to individual HFEA records. In collaboration with colleagues at University College London, a definitive study was undertaken^70^ to establish whether children born between 1992 and 2008 following IVF type procedures were at an increased childhood cancer risk when compared to their naturally conceived peers. Overall, children born following these procedures were not found to be at increased risk of developing childhood cancer. Further regulation required the analysis of outcomes for children born following interventions involving donor eggs, sperm or embryos to be undertaken separately. However, again, no increased risk of overall childhood cancer was found.^71^ In two papers, analysing cancer incidence among 26,692 children who were born after IVF during the years 1982–2005 in Sweden an increased risk of Langerhans cell histiocytosis was found.^72,73^ A collaborative UK study describing the incidence of this condition in children born following assisted reproduction procedures is nearing completion.

#### Birth factors

A case–control study of the relations between childhood cancers, parental age and sibship position was carried out in collaboration with OPCS.^74^ For this study, it was necessary to have temporary access to the confidential birth records held at OPCS, to enable linkage be carried out between these records and those held by CCRG. Only anonymised records could be released after the linkage process. The most striking findings from this study of 10,162 matched pairs were that the risk of ALL decreased with increasing parity and increased with increasing parental age; the latter effect was not explained by the association with Down syndrome. A predicted association between high paternal age and new germ cell mutations in retinoblastoma was also found, though this was not statistically significant. The corresponding result for maternal age was in fact rather more marked, though because of the strong correlation between paternal and maternal age it was not possible to disentangle these effects.

Clinically recorded birthweight was appended to the birth registration details of nearly all children born in England and Wales from about 1980. This made possible some of the largest studies of intrauterine growth and childhood cancer risk^75–77^ which, taken as a whole, showed birthweight to be associated with risk for approximately half of all childhood cancers. Increasing birthweight raises the risk most notably of leukaemia, tumours of the central nervous system, renal tumours and soft-tissue sarcomas. Associations were also observed between high birthweight and the risk of neuroblastoma, lymphoma, germ cell tumours and malignant melanomas. By contrast, increasing birthweight reduces the risk of hepatic tumours. No association was observed between birthweight and the risk of retinoblastoma or bone tumours. Results for US datasets were very similar to those for the UK(NRCT).

A job exposure matrix approach, based on the details of father’s occupation provided at birth registration, was used to study paternal occupational exposures for the majority of childhood cancers registered by the NRCT.^78–82^ Overall, these studies showed little, if any, support for the hypothesis that paternal occupational exposure is an important aetiological factor for childhood cancer. Analysis of leukaemia cases, however, showed some evidence of a positive association with paternal occupations involving social contact; in addition, higher paternal occupational social class was associated with increased lymphoid leukaemia risk.^78^

#### Socioeconomic factors

There have been various reports of an association between socioeconomic status and the incidence of childhood malignant disease. For childhood ALL, persistence of a small socioeconomic gradient reported in previous studies was confirmed by Kroll et al.^83^ A study in which the NRCT was linked to clinical trials data was consistent with this gradient being partly due to systematic under-diagnosis in less affluent communities in Britain during the 1980s and 1990s.^84^ The authors concluded “Under-diagnosis in poorer communities may have contributed to socioeconomic variation in recorded childhood acute lymphoblastic leukaemia incidence within Great Britain, and elsewhere” They also stated that their findings for ALL were consistent with the “pre-emptive infection hypothesis”, which proposes that some children with leukaemia die from infection without leukaemia being suspected. The authors referred to Stewart^85^ who seems to have been the first to propose this hypothesis.

#### Geographical studies

The CCRG focus on childhood cancer and leukaemia around nuclear installations stimulated geographical analyses of the NRCT data, which were enhanced by accurate geo-referencing of addresses at birth and registration. Reports of aggregations of cases in a number of areas were examined, including Camelford, Cornwall following a water pollution incident,^86^ and Baglan Bay, Wales following concern about the incidence of leukaemia and lymphoma in children and young people.^87^ None of these investigations led to a clear causal connection with any putative risk mechanism, but they nevertheless have considerable socio-political value for allaying anxieties about environmental hazards.

The problem of assessing such “clusters” of cases is exacerbated by the widely believed hypothesis that cases tend to cluster naturally. Considerable effort has been expended on exploring this hypothesis, particularly in view of the possibility that clustering might be indicative of a contagious mechanism.

Because of the widespread interest in geographical variation, the CCRG made a standard dataset on childhood leukaemia and non-Hodgkin lymphoma (NHL) available to a number of other research groups, in order for them to explore a variety of analytical methods for the examination of incidence rates and the detection of clustering. The results were collated and published in an OPCS volume.^88^

Comprehensive geographical analyses of NRCT data are reported in COMARE’s 11th Report^89^ and further described in a series of papers in collaboration with colleagues in the University of Newcastle.^90,91^ NRCT data were also used for EUROCLUS, a collaborative European study of the issues.^92^ Certainly there is evidence of some variation in incidence of childhood leukaemia, but this is largely related to differences between districts with varying socioeconomic characteristics (see above). Kinlen’s “population-mixing” hypothesis, linking risk of childhood leukaemia to herd immunity, was investigated in a series of collaborative studies using NRCT data.^93–96^ A recent review of leukaemia clustering is given in Bithell.^97^

Geographical data were later made available to colleagues in the University of Newcastle for collaborative studies of population mixing^98^ and the possible hazard of proximity to railway lines^99^; in the former paper an increase in leukaemia was detected in urban areas with high levels of inward migration; in the latter a very weak effect of proximity could be attributed to two urban wards, possibly affected by the population mixing phenomenon. The possibility of a common aetiology for different tumours was explored in a further collaborative paper on the cross-space-time-clustering of places and dates of birth and diagnosis; two links involving Hodgkin lymphoma were regarded as marginally significant after allowing for multiple testing.^100^

The NRCT has the residential addresses at both birth and diagnosis for cases. An investigation quantified the relationship between residence at birth and diagnosis^101^ About half the cases were still at the birth location at diagnosis and those who had moved had usually not moved far. This is important in interpreting aetiological studies in which exposures to the agents of interest vary with location

Swanson et al.^102^ used NRCT data in an investigation of the precision of various sources of such location information, particularly in the context of studies of non-ionising radiation.

### Studies of effects on clinical practice

Throughout its existence, the CCRG carried out population-based studies of aspects of clinical service delivery such as type and caseload of treating hospital and entry to clinical trials for a wide range of childhood cancers, usually in relation to outcome. This work began with retinoblastoma^103,104^ and continued with studies of Wilms tumour,^105,106^ leukaemia^107–110^ and bone tumours^111^ among others. Many of these studies revealed survival advantages associated with treatment at large specialist centres or inclusion in clinical trials. (Though a study of early mortality following surgery for childhood brain tumours demonstrated an absence of such an effect.^112^) Pritchard et al.^106^ showed that there was a risk of over-treatment for patients with Wilms’ tumour treated outside paediatric oncology centres.

We believe that these studies on the effects of entry to clinical trials and on centralisation of clinical care have had an impact on clinical practice.

### Facilitating studies of clinical data

As already indicated, one of the most important aspects of the CCRG’s work was the extensive series of collaborative studies with other organisations and individuals. Many analyses based wholly or in part on NRCT data were carried out by, or in collaboration with, the UKCCSG/CCLG, individual clinicians and pathologists, and clinical trial organisers. Sets of records or the results of analyses relating to particular diagnostic groups or to groups of cases in particular hospitals or areas were made available to paediatric oncologists and other research workers. The subjects of these studies included tumours of the kidney^113,114^ and ovary,^115^ cardiac tumours,^116^ leukaemia,^117^ carcinomas,^118^ soft-tissue sarcoma^119,120^ and NHL.^121,122^

A national registry of paediatric myelodysplasia was set up and analysed in collaboration with colleagues at Great Ormond Street Hospital.^123^

### International collaborative studies

Most of the collaborative studies referred to above involved epidemiologists and clinicians within the UK. There were also two groups of international collaborative studies:

#### International Agency for Research on Cancer

The CCRG initiated, and was a leading collaborator in, the study “International Incidence of Childhood Cancer” based at the International Agency for Research on Cancer (IARC), which has resulted in two monographs^124,125^ with a third volume in preparation, a series of papers on the same topic^126–128^ and successive editions of the standard classification system for childhood tumours.^129–131^ Other IARC projects in which CCRG played an important part were a study of leukaemia incidence following the Chernobyl accident^132,133^ and the Automated Childhood Cancer Information System (ACCIS), concerned with cancer incidence and survival among children and adolescents throughout Europe, for which the initial paper^134^ was followed by a monographic special issue of *European Journal of Cancer*^135^ containing 19 papers, nine of them involving CCRG authors.

#### International collaborative studies of childhood cancer survival

The CCRG participated in all analyses of childhood cancer survival within the Europe-wide EUROCARE collaboration.^136–141^ Most recently, the NRCT contributed data and expertise to the CONCORD study of worldwide cancer survival.^142,143^

## Discussion

### Overview of research and findings

The NRCT, and work of the CCRG based upon it, has been central in developing understanding of patterns and trends in childhood cancer and its treatment. Work on retinoblastoma has been a particular focus. Studies of familial factors have also been important. A major area of work has been aetiological studies where important contributions have been made to our understanding of risks associated, for example, with both ionising and non-ionising radiation and with assisted reproductive technology. “Clusters” of childhood cancer have attracted great public interest and work based on the NRCT has been vital in investigating this topic. The success of the first volume of *International Incidence of Childhood Cancer* and the suite of papers based on those data paved the way for CCRG involvement in other international collaborations both at IARC and elsewhere.

### Research resources

The main research resource developed by the Group was the computerised NRCT database and a large series of associated paper records and digitised images of many of these. Most of the work described above was based on these electronic and paper records. In addition, in the course of both the OSCC and the routine data collection of the NRCT a series of records relating to family histories generally, and to sibs and twins in particular has been assembled; there is in particular an especially large set of pedigree data relating to retinoblastoma. The data are archived; we are attempting to ensure that, subject to the usual restrictions and permissions concerning access to confidential data (Research Ethical Committees; the Confidentiality Advisory Group), they can be made available for specified research projects.

Another major resource was an electronic database containing bibliographic details of around 16,000 references related to childhood cancer; many of these include electronic links to the actual publications.

We hope that one consequence of the publication of this paper and the two accompanying ones will be to draw attention to the existence of these resources.

## Electronic supplementary material

Supplementary information is available for this paper at https://doi.org/10.1038/s41416-018-0181-z. This is a file of CCRG Publications on Childhood Cancer Research

## Data Availability

This paper did not involve any new collection or analyses of data. Much of the work described was based on the UK National Registry of Childhood Tumours. Our hope is that these data will be available for future analyses. This is discussed in the section “Research Resources” above.

## Electronic supplementary material

**Supplementary information** available for this paper at 10.1038/s41416-018-0181-z. This is a file of CCRG Publications on Childhood Cancer
