# Supplementary material for: Childhood cancer research in Oxford II: The Childhood Cancer Research Group
Source: Br J Cancer. 2018 Aug 21;119(6):763–70. doi: 10.1038/s41416-018-0181-z (PMC6173767; doi:10.1038/s41416-018-0181-z)
Supplement: Supplementary file 1 — Supplementary information is available for this paper at https://doi.org/10.1038/s41416-018-0181-z. This is a file of CCRG Publications on Childhood Cancer Research [file 41416_2018_181_MOESM1_ESM.rtf]

Alexander FE, Boyle P, Carli PM, Coebergh JW, Draper GJ, Ekbom A, Levi F, McKinney P, McWhirter W, Michaelis J, Peris-Bonet R, Petridou E, Pompe-Kirn V, Plisko I, Pukkala E, Rahu M, Storm H, Terracini B, Vatten L, Wray N (1998) Spatial clustering of childhood leukaemia: summary results from the EUROCLUS project. Br J Cancer 77(5): 818-824

Alexander FE, Boyle P, Carli PM, Coebergh JW, Draper GJ, Ekbom A, Levi F, McKinney PA, McWhirter W, Magnani C, Michaelis J, Olsen JH, Peris-Bonet R, Petridou E, Pukkala E, Vatten L (1998) Spatial temporal patterns in childhood leukaemia: further evidence for an infectious origin. EUROCLUS project. Br J Cancer 77(5): 812-817

Alexander FE, Boyle P, Carli PM, Coebergh JW, Ekbom A, Levi F, McKinney PA, McWhirter W, Michaelis J, Peris-Bonet R, Petridou E, Pompe-Kirn V, Plesko I, Pukkala E, Rahu M, Stiller CA, Storm H, Terracini B, Vatten L, Wray N (1999) Population density and childhood leukaemia:  Results of the EUROCLUS study. European journal of cancer (Oxford, England : 1990) 35(3): 439-444

Alexander FE, Wray N, Boyle P, Bring J, Coebergh JW, Draper GJ, Levi F, McKinney PA, Michaelis J, Peris-Bonet R, Petridou E, Pukkala E, Storm H, Terracini B, Vatten L, on behalf of the EUROCLUS project (1996) Clustering of childhood leukaemia: a European study in progress. J Epidemiol Biostat 1: 13-24

Allemani C, Weir HK, Carreira H, Harewood R, Spika D, Wang XS, Bannon F, Ahn JV, Johnson CJ, Bonaventure A, Marcos-Gragera R, Stiller C, Azevedo e Silva G, Chen WQ, Ogunbiyi OJ, Rachet B, Soeberg MJ, You H, Matsuda T, Bielska-Lasota M, Storm H, Tucker TC, Coleman MP (2015) Global surveillance of cancer survival 1995-2009: analysis of individual data for 25,676,887 patients from 279 population-based registries in 67 countries (CONCORD-2). Lancet 385(9972): 977-1010

Anoop P, Sankpal S, Stiller C, Tewari S, Lancaster DL, Khabra K, Taj M (2012) Outcome of childhood relapsed or refractory mature B-cell non-Hodgkin lymphoma and acute lymphoblastic leukaemia. Leuk Lymphoma 53(10): 1882-1888

Arndt V, Lacour B, Steliarova-Foucher E, Spix C, Znaor A, Pastore G, Stiller C, Brenner H (2007) Up-to-date monitoring of childhood cancer long-term survival in Europe: tumours of the sympathetic nervous system, retinoblastoma, renal and bone tumours, and soft tissue sarcomas. Annals of oncology 18(10): 1722-1733

Arrundale J, Bain M, Botting B, Brewster D, Cartwright R, Chalmers J, Coggan D, Elliott P, Jackson I, McKinney T, McNally R, Miles DPB, Quinn MJ, Sharp L, Staines A, Stiller C, Wilkinson P (1997) Handbook And Guide To The Investigation Of Clusters Of Diseases. University of Leeds: Leukaemia Research Fund Centre for Clinical Epidemiology


Barnes WSF, Craft AW, Hunter AS, Stiller CA (1986) Primary malignant cardiac tumors in children. Pediatr Hematol Oncol 3(4): 347-351

Benjamin S, Kroll ME, Cartwright RA, Clough JV, Gorst DW, Proctor SJ, Ross JRY, Taylor PRA, Wheatley K, Whittaker JA, Stiller CA (2000) Haematologists' approaches to the management of adolescents and young adults with acute leukaemia. Br J Haematol 111(4): 1045-1050

Berrington de Gonzalez A, Salotti JA, McHugh K, Little MP, Harbron RW, Lee C, Ntowe E, Braganza MZ, Parker L, Rajaraman P, Stiller C, Stewart DR, Craft AW, Pearce MS (2016) Relationship between paediatric CT scans and subsequent risk of leukaemia and brain tumours: assessment of the impact of underlying conditions. Br J Cancer 114(4): 388-94

Birch JM, Hartley AL, Teare MD, Blair V, McKinney PA, Mann JR, Stiller CA, Draper GJ, Johnston HE, Cartwright RA, Waterhouse JAH (1990) The Inter-Regional Epidemiological Study of Childhood Cancer (IRESCC): case-control study of children with central nervous system tumours. Br J Neurosurg 4(1): 17-26

Birch JM, Mann JR, Cartwright RA, Draper GJ, Waterhouse JAH, Hartley AL, Johnston HE, McKinney PA, Stiller CA, Hopton PA, for the IRESCC Group (1985) The Inter-Regional Epidemiological Study of Childhood Cancer (IRESCO). Study design, control selection and data collection. Br J Cancer 52(6): 915-922

Bithell JF (2010) Childhood cancer and proximity to mobile phone masts. Epidemiological studies show no increased risk. BMJ 341: 1-2

Bithell JF, Draper GJ (1995) Apparent association between benzene and childhood leukaemia: methodological doubts concerning a report by Knox. J Epidemiol Community Health 49(4): 437-439

Bithell JF, Draper GJ (1999) Uranium-235 and childhood leukaemia around Greenham Common airfield. J Radiol Prot 19(3): 253-259

Bithell JF, Dutton SJ, Draper GJ, Neary NM (1994) Distribution of childhood leukaemias and non-Hodgkin's lymphomas near nuclear installations in England and Wales. BMJ 309(6953): 501-505

Bithell JF, Dutton SJ, Neary NM, Vincent TJ (1995) Controlling for socioeconomic confounding using regression methods. J Epidemiol Community Health 49(Suppl 2): S15-S19

Bithell JF, Keegan TJ, Kroll ME, Murphy MFG, Vincent TJ (2008) Childhood leukaemia near British Nuclear installations: methodological issues and recent results. Radiat Prot Dosimetry 132(2): 191-197

Bithell JF, Keegan TJ, Kroll ME, Murphy MFG, Vincent TJ (2010) Response to Korblein and Fairlie: Correction and extensions to the calculation in "childhood leukaemia near British Nuclear Installations: Methodology issues and recent results". Radiat Prot Dosimetry 138(1): 89-91

Bithell JF, Murphy MFG, Stiller CA, Toumpakari E, Vincent T, Wakeford R (2013) Leukaemia in young children in the vicinity of British nuclear power plants: a case-control study. Br J Cancer 109(11): 2880-2885

Bithell JF, Stewart AM (1975) Pre-natal irradiation and childhood malignancy: a review of British data from the Oxford Survey. Br J Cancer 31(3): 271-287

Bithell JF, Stiller CA (1977) Dose-Response analysis of case-control data: The carcinogenic risk of obstetric irradiation. In Proceedings of a Symposium, University of Bremen., Behnen., Kinder., Osius., Schafer., Timm. (eds), pp 168-171. Bremen: 

Bithell JF, Stiller CA (1988) A new calculation of the carcinogenic risk of obstetric X-raying. Statistics in medicine 7(8): 857-864

Bithell JF, Upton RG (1976) A mixed model for survival applied to British children with neuroblastoma. In Proceedings of the European Congress of Statisticians, Grenoble., Barra JR (ed), pp 635-646. Amsterdam: North Holland Publishing Company

Bithell JF, Vincent TJ (2000) Geographical variations in childhood leukaemia incidence. In Spatial Epidemiology, Elliott P, Wakefield JC, Best NG, Briggs DJ (eds), Chapter 18, pp 317-332. Oxford: Oxford University Press

Black RJ, Urquhart JD, Kendrick SW, Bunch KJ, Warner J, Adams Jones D (1992) Incidence of leukaemia and other cancers in birth and schools cohorts in the Dounreay area. BMJ 304(6839): 1401-1405

Blakey K, Feltbower RG, Parslow RC, James PW, Gómez Pozo B, Stiller C, Vincent TJ, Norman P, McKinney PA, Murphy MF, Craft AW, McNally RJQ (2014) Is fluoride a risk factor for bone cancer? Small area analysis of osteosarcoma and Ewing sarcoma diagnosed among 0–49-year-olds in Great Britain, 1980–2005. International Journal of Epidemiology 43(1): 224-234

Blot WJ, Draper GJ, Kinlen L, Kinnier Wilson LM (1980) Childhood cancer in relation to prenatal exposure to chickenpox. Br J Cancer 42(2): 342-344

Blot WJ, Stiller CA, Kinnier Wilson LM (1980) Oral clefts and childhood cancer. Lancet 315(8170): 722

Bonaventure A, Harewood R, Stiller CA, Gatta G, Clavel J, Stefan DC, Carreira H, Spika D, Marcos-Gragera R, Peris-Bonet R, Pineros M, Sant M, Kuehni CE, Murphy MFG, Coleman MP, Allemani C (2017) Worldwide comparison of survival from childhood leukaemia for 1995-2009, by subtype, age, and sex (CONCORD-2): a population-based study of individual data for 89 828 children from 198 registries in 53 countries. The Lancet Haematology 4(5): e202-e217

Brada M, Hawkins MM (1995) Brain tumours in children - lifetime for patients and investigators. Int J Radiat Oncol Biol Phys 31(3): 671-672

Brennan B, Stevens M, Kelsey A, Stiller CA (2010) Synovial sarcoma in childhood and adolescence: A retrospective series of 77 patients registered by the Children's Cancer and Leukaemia Group between 1991 and 2006. Pediatr Blood Cancer 55(1): 85-90

Brennan B, Stiller C (2010) Rare tumours. In Pediatric Hematology and Oncology. Scientific Principles and Clinical Practice, Estlin EJ, Gilbertson RJ, Wynn RF (eds), Chapter 19, pp 319-332. Wiley-Blackwell

Brennan B, Stiller C, Bourdeaut F (2013) Extracranial rhabdoid tumours: what we have learned so far and future directions. The Lancet Oncology 14(8): e329-e336

Brennan BMD, Foot ABM, Stiller C, Kelsey A, Vujanic G, Grundy R, Pritchard-Jones K, on behalf of the United Kingdom Children's Cancer Study Group (UKCCSG) (2004) Where to next with extracranial rhabdoid tumours in children. European journal of cancer (Oxford, England : 1990) 40(4): 624-626

Breslow NE, Lange JM, Friedman DL, Green DM, Hawkins MM, Murphy MFG, Neglia JP, Olsen JH, Peterson SM, Stiller CA, Robison LL (2010) Secondary malignant neoplasms following Wilms Tumor: an International Collaborative Study. Int J Cancer 127(3): 657-666

Buckley JD, Buckley CM, Breslow NE, Draper GJ, Roberson PK, Mack TM (1996) Concordance for childhood cancer in twins. Med Pediatr Oncol 26(4): 223-229

Bunch KJ, Muirhead CR, Draper GJ, Hunter N, Kendall GM, O'Hagan JA, Phillipson MA, Vincent TJ, Zhang W (2009) Cancer in the offspring of female radiation workers: a record linkage study. Br J Cancer 100(1): 213-218

Bunch KJ, Swanson J, Vincent TJ, Murphy MF (2015) Magnetic fields and childhood cancer: an epidemiological investigation of the effects of high-voltage underground cables. J Radiol Prot 35(3): 695-705

Bunch KJ, Swanson J, Vincent TJ, Murphy MF (2016) Epidemiological study of power lines and childhood cancer in the UK: further analyses. J Radiol Prot 36(3): 437-455

Bunch KJ, Vincent TJ, Black RJ, Pearce MS, McNally RJQ, McKinney PA, Parker L, W. CA, Murphy MFG (2014) Updated investigations of cancer excesses in individuals born or resident in the vicinity of Sellafield and Dounreay. Br J Cancer 111: 1814–1823

Butland BK, Muirhead CR, Draper GJ (1990) Radon and leukaemia. Lancet 335(8701): 1338-1339

Cartwright RA, McKinney PA, Hopton PA, Birch JM, Hartley AL, Mann JR, Waterhouse JAH, Johnston HE, Draper GJ, Stiller CA (1984) Ultrasound examinations in pregnancy and childhood cancer. Lancet 324(8410): 999-1000

Cartwright RA, McNally RJQ, Rowland DJ, Staines A, Stiller C (1997) Malignancies in the adolescent. In The Descriptive Epidemiology of Leukaemia and Related Conditions in Parts of the United Kingdom 1984-1993, Cartwright RA, McNally RJQ, Rowland DJ, Thomas J, Staines A, Stiller C (eds), Chapter 3, pp 46-55 and CD-Rom. London: Leukaemia Research Fund

Cartwright RA, McNally RJQ, Rowland DJ, Thomas J, Stiller C (1997) Introduction to the Data Collection Study. In The Descriptive Epidemiology of Leukaemia and Related Conditions in Parts of the United Kingdom 1984-1993, Cartwright RA, McNally RJQ, Thomas J, Staines A, Stiller C (eds), Chapter 1, pp 1-8. London: Leukaemia Research Fund

Chernyavskiy P, Kendall GM, Wakeford R, Little MP (2016) Spatial prediction of naturally occurring gamma radiation in Great Britain. Journal of Environmental Radioactivity 164: 300-311

Chumas P, Kenny T, Stiller C (2011) Subspecialisation in neurosurgery-does size matter? Acta Neurochir (Wien) 153(6): 1231-1236

Coebergh JWW, Capocaccia R, Gatta G, Magnani C, Stiller CA (2001) Childhood cancer survival in Europe, 1978-1992:  the EUROCARE study. European journal of cancer (Oxford, England : 1990) 37(6): 671-672

Craft AW, Parker L, Stiller CA, Cole M (1995) Screening for Wilms' tumour in patients with aniridia, Beckwith syndrome or hemihypertrophy. Med Pediatr Oncol 24(4): 231-234

Crocetti E, Trama A, Stiller C, Caldarella A, Soffietti R, Jaal J, Weber DC, Ricardi U, Slowinski J, Brandes A, RARECARE working group (2012) Epidemiology of glial and non-glial brain tumours in Europe. European journal of cancer (Oxford, England : 1990) 48(10): 1532-1542


de Vathaire F, Hardiman C, Shamsaldin A, Campbell S, Grimaud E, Hawkins M, Raquin M, Oberlin O, Diallo I, Zucker J-M, Panis X, Lagrange JL, Daly-Schveitzer N, Lemerle J, Chavaudra J, Schlumberger M, Bonaiti C (1999) Thyroid carcinomas after irradiation for a first cancer during childhood. Arch Intern Med 159(22): 2713-2719

de Vathaire F, Hawkins M, Campbell S, Oberlin O, Raquin M-A, Schlienger J-Y, Shamsaldin A, Diallo I, Bell J, Grimaud E, Hardiman C, Lagrange J-L, Daly-Schveitzer N, Panis X, Zucker J-M, Sancho-Garnier H, Eschwège F, Chavaudra J, Lemerle J (1999) Second malignant neoplasms after a first cancer in childhood: temporal pattern of risk according to type of treatment. Br J Cancer 79(11/12): 1884-1893

de Vathaire F, Shamsaldin A, Grimaud E, Campbell S, Guerra M, Raquin M, Bessa E, Hardiman C, Jan P, Rumeau N, Diallo I, Nicolazic G, Lamon A, Oberlin O, de Cervens C, Suarez A, Meresse V, Hawkins MM, Bell J, Schlienger J-Y, Lagrange J-L, Gaboriaud G, Daly-Schveitzer N, Panis X, Zucker J-M, Lermerle J, Eschwege F, Sancho-Garnier H, Chavaudra J (1995) Solid malignant neoplasms after childhood irradiation:  decrease of the relative risk with time after irradiation. Comptes rendus de l'Académie des sciencesSérie III, Sciences de la vie 318(4): 483-490

Dockerty JD, Draper GJ, Vincent TJ, Rowan SD, Bunch KJ (2001) Case-control study of parental age, parity and socioeconomic level in relation to childhood cancers. Int J Epidemiol 30(6): 1428-1437

Donaldson SS, Draper GJ, Flamant F, Gerard-Marchant R, Mouriesse H, Newton WA, Lemerle J (1986) Topography of childhood tumors: pediatric coding system. Pediatr Hematol Oncol 3(3): 249-258

dos Santos Silva I, Swerdlow AJ, Stiller CA, Reid A (1999) Incidence of testicular germ-cell malignancies in England and Wales:  trends in children compared with adults. Int J Cancer 83(5): 630-634

Doyle P, Bunch KJ, Beral V, Draper GJ (1998) Cancer incidence in children conceived with assisted reproduction technology. Lancet 352(9126): 452-453

Draper G, Vincent T, Kroll ME, Swanson J (2005) Childhood cancer in relation to distance from high voltage power lines in England and Wales: a case-control study. BMJ 330(7503): 1290-1294

Draper GJ (1977) Epidemiological studies and the genetic component in the aetiology of childhood cancers. In Tumours of Early Life in Man and Animals, the Proceedings of the VIth Perugia Quadrennial International Conference on Cancer., Severi (ed), pp 101-112. Perugia: 

Draper GJ (1980) Population studies of incidence, survival and follow-up. In Status of the Curability of Childhood Cancers, 24th M.D. Anderson Clinical Conference on Cancer., van Eys J, Sullivan MP (eds), pp 285-294. New York: Raven Press

Draper GJ (1985) The aetiology and epidemiology of bone and soft-tissue sarcomas. In Bone Tumours and Soft-Tissue Sarcomas, D'Angio GJ, Evans AE (eds), Chapter Chapter 1, pp 1-13. London: Edward Arnold

Draper GJ (1985) Genetic aspects of the aetiology of childhood cancer. In Familial Cancer, Muller HJ, Weber W (eds), pp 139-141. Basel: Karger

Draper GJ (1985) Statistical methods for the analysis of epidemiological studies of familial cancer. In Familial Cancer, Muller HJ, Weber W (eds), pp 199-201. Basel: Karger


Draper GJ (1987) Cancer incidence in the relatives of children with cancer. In Lectures and Symposia of the 14th International Cancer Congress, Budapest., Lapis K, Eckhardt S (eds) Vol. 8, pp 253-260. Budapest: 

Draper GJ (1988) Childhood leukaemia and Dounreay. Lancet 332(8605): 287

Draper GJ (1988) Screening for neuroblastoma.  Japanese studies suggest that it may save lives. BMJ 297(6642): 152-153

Draper GJ (1989) General overview of studies of multigeneration carcinogenesis in man, particularly in relation to exposure to chemicals. In Perinatal and Multigeneration Carcinogenesis, IARC Scientific Publications No.96, Napalkov NP, Rice J, M., Tomatis L, Yamasaki H (eds), pp 275-288. Lyon: International Agency for Research on Cancer

Draper GJ (1990) Childhood cancer in Zaire. Lancet 335(8688): 553-554

Draper GJ (1990) The National Registry of Childhood Tumours and its uses in epidemiological studies of radiation risks. Radiol Prot Bull 113: 5-8

Draper GJ (1990) Studies on the possible role of preconception exposure to radiation in human carcinogenesis. Radiat Res 124(3): 364-365

Draper GJ (1991) The geographical distribution of childhood leukaemia and non-Hodgkin lymphomas in the United Kingdom. In Data Requirements and Methods for Analysing Spatial Patterns of Disease in Small Areas, pp 115-117. Copenhagen: WHO Regional Office for Europe

Draper GJ (1992) An overview of reports and current research concerning childhood leukaemia and cancer around nuclear installations in the UK. The Science of the total environment 127(1-2): 9-12

Draper GJ (1992) A review of the epidemiological studies of childhood leukemia around nuclear facilities in the United Kingdom. In Symposium on Leukaemia Clustering, Ottawa, Canada  March 11th, 1992   Proceedings, Elaguppillai V, Goyette JP, Hill G, Krewski D, Osborne RV (eds), pp 13-18. Ottawa: Atomic Energy Control Board

Draper GJ (1993) Electromagnetic fields and childhood cancer.  No causal relation has been established. BMJ 307(6909): 884-885

Draper GJ (1993) Familial aggregation of childhood leukaemias and lymphomas. In Epidemiology of Childhood Leukaemia, Michaelis J (ed), pp 223-233. Stuttgart: Gustav Fischer Verlag

Draper GJ (1995) Cancer. In The Health of Our Children, Botting B (ed), Chapter 10, pp 135-147. London: HMSO

Draper GJ (1995) Childhood cancer: trends in incidence, survival and mortality. European journal of cancer (Oxford, England : 1990) 31A(5): 653-654

Draper GJ (1997) The analysis of registry data in relation to various different types of hypothesis regarding the geographical distribution of disease. Cent Eur J Public Health 5(2): 90-92

Draper GJ, Birch JM, Bithell JF, Kinnier Wilson LM, Leck I, Marsden HB, Morris Jones PH, Stiller CA, Swindell R (1982) Childhood cancer in Britain: Incidence, mortality and survival, OPCS Studies on Medical and Population Subjects No 37. London: HMSO

Draper GJ, Bower BD, Darby SC, Doll R (1989) Completeness of registration of childhood leukaemia near nuclear installations and elsewhere in the Oxford region. BMJ 299(6705): 952


Draper GJ, Elliott P (1991) Variations in incidence rates and factors affecting them - summary. In The Geographic Epidemiology of Childhood Leukaemia and non-Hodgkin Lymphomas in Great Britain, 1966-83, OPCS Studies on Medical and Population Subjects  no.53, Draper GJ (ed), Chapter 7, pp 57-59. London: HMSO

Draper GJ, Heaf MM, Kinnier Wilson LM (1977) Occurrence of childhood cancers among sibs and estimation of familial risks. J Med Genet 14(2): 81-90

Draper GJ, Kendall GM, Muirhead CR, Sorahan T, Fox AJ, Kinlen LJ (1992) Cancer in the children of radiation workers. Radiol Prot Bull 129: 10-14

Draper GJ, Kroll ME, Stiller CA (1994) Childhood Cancer. In Trends in Cancer Incidence and Mortality, Doll R, Fraumeni JF, Muir CS (eds), Cancer Surveys vol 19/20 edn, pp 493-517. 

Draper GJ, Little MP, Sorahan T, Kinlen LJ (1998) Cancer in the offspring of radiation workers - authors' reply. BMJ 316(7145): 1673

Draper GJ, Little MP, Sorahan T, Kinlen LJ, Bunch KJ, Conquest AJ, Kendall GM, Kneale GW, Lancashire RJ, Muirhead CR, O'Connor CM, Vincent TJ (1997) Cancer in the offspring of radiation workers: a record linkage study. BMJ 315(7117): 1181-1188

Draper GJ, Little MP, Sorahan T, Kinlen LJ, Bunch KJ, Conquest AJ, Kendall GM, Kneale GW, Lancashire RJ, Muirhead CR, O'Connor CM, Vincent TJ, Thomas JM, Goodill AA, Vokes J, Haylock RGE (1997) NRPB-R298: Cancer in the Offspring of Radiation Workers - a Record Linkage Study, NRPB-R298 edn. Didcot: NRPB

Draper GJ, McNinch A (1994) Vitamin K for neonates: the controversy. BMJ 308(6933): 867-868

Draper GJ, Parkin DM (1992) Cancer incidence data for children. In Geographical and Environmental Epidemiology: Methods for Small - Area studies, Elliott P, Cuzick J, English D, Stern R (eds), Chapter 6, pp 63-71. London: Oxford University Press

Draper GJ, Passmore SJ (1995) Current UK studies on the possible association between vitamin K and childhood cancer. In Vitamin K in Infancy, Sutor AH, Hathaway WE (eds), pp 321-326. Stuttgart: Schattauer

Draper GJ, Sanders BM, Brownbill PA, Hawkins MM (1992) Patterns of risk of hereditary retinoblastoma and applications to genetic counselling. Br J Cancer 66(1): 211-219

Draper GJ, Sanders BM, Kingston JE (1986) Second primary neoplasms in patients with retinoblastoma. Br J Cancer 53(5): 661-671

Draper GJ, Sanders BM, Lennox EL, Brownbill PA (1996) Patterns of childhood cancer among siblings. Br J Cancer 74(1): 152-158

Draper GJ, Stiller CA (1989) Cautious optimism. Paediatr Nurs 1: 22-24

Draper GJ, Stiller CA (1989) Screening for neuroblastoma. Lancet 333(8629): 98

Draper GJ, Stiller CA (1992) Intramuscular vitamin K and childhood cancer. BMJ 305(6855): 709

Draper GJ, Stiller CA, Cartwright RA, Craft AW, Vincent TJ (1993) Cancer in Cumbria and in the vicinity of the Sellafield nuclear installation, 1963-90. BMJ 306(6870): 89-94

Draper GJ, Stiller CA, Fearnley H, Lennox EL, Roberts EM, Sanders BM (1988) United Kingdom - England and Wales.  National registry of childhood tumours, 1971-1980. In International incidence of childhood cancer, Parkin DM, Stiller CA, Draper GJ, Bieber CA, Terracini B, Young JL (eds), 87 edn, pp 295-298. Lyon: IARC Scientific Publications

Draper GJ, Stiller CA, O'Connor CM, Vincent TJ (1991) Introduction and objectives. In The Geographical Epidemiology of Childhood Leukaemia and Non-Hodgkin Lymphomas in Great Britain, 1966-83,OPCS Studies on Medical and Population Subjects No.53, Draper GJ (ed), Chapter 1, pp 1-6. London: HMSO

Draper GJ, Stiller CA, O'Connor CM, Vincent TJ, Elliott P, McGale P, Rodrigues L, Hills M, Black RJ, Sharp L, Urquhart JD, Alexander FE, Gilman EA, Knox EG, Besag J, Newell J, Craft A, Openshaw S, Cuzick J, Gardner MJ (1991) The Geographical Epidemiology of Childhood Leukaemia and Non-Hodgkin Lymphomas in Great Britain, 1966-83, OPCS Studies on Medical and Population Subjects No.53. London: OPCS

Draper GJ, Vincent TJ (1997) Death rates from childhood leukaemia near nuclear sites.  Findings were probably due to chance fluctuations in small numbers of deaths. BMJ 315(7117): 1233

Draper GJ, Vincent TJ, Kroll ME, Swanson J (2005) Childhood cancer and powerlines: Author's reply. BMJ 331(7517): 636-637

Draper GJ, Vincent TJ, O'Connor CM, Stiller CA (1991) Socio-Economic factors and variations in incidence rates between county districts. In The Geographical Epidemiology of Childhood Leukaemia and Non-Hodgkin Lymphomas in Great Britain 1966-83, OPCS Studies on Medical and Population Subjects No 53, Draper GJ (ed), Chapter 5, pp 37-45. London: HMSO

Eden OB, Stiller CA, Gerrard MP (1988) Improved survival for childhood acute lymphoblastic leukemia: possible effect of protocol compliance. Pediatr Hematol Oncol 5(2): 83-91

ELF-EMF European Feasibility Study Group (1997) Need for a European approach to the effects of extremely low-frequency electromagnetic fields on cancer. Scand J Work Environ Health 23(1): 5-14

Elliott P, McGale P, Vincent TJ (1991) Description of population data and definitions of areas. In The Geographical Epidemiology of Childhood Leukaemia and Non-Hodgkin Lymphomas in Great Britain, 1966-83,OPCS Studies on Medical and Population Subjects No.53, Draper GJ (ed), Chapter 3, pp 17-23. London: HMSO

England RJ, Haider N, Vujanic GM, Kelsey A, Stiller CA, Pritchard-Jones K, Powis M (2011) Mesoblastic Nephroma: A Report of the United Kingdom Children's Cancer and Leukaemia Group (CCLG). Pediatr Blood Cancer 56(5): 744-748

Fear NT, Vincent TJ, King JC, MacCarthy A, Bunch KJ, Murphy MFG (2009) Wilms Tumour and Paternal Occupation: An Analysis of Data From the National Registry of Childhood Tumours. Pediatr Blood Cancer 53(1): 28-32

Fletcher EWL, Baum JD, Draper GJ (1986) The risk of diagnostic radiation of the newborn. Br J Radiol 59(698): 165-170

Gao W, Verne J, Peacock J, Stiller C, Wells C, Greenough A, Higginson IJ (2016) Place of death in children and young people with cancer and implications for end of life care: a population-based study in England, 1993-2014. BMC cancer 16(1): 727

Gatta G, Botta L, Rossi S, Aareleid T, Bielska-Lasota M, Clavel J, Dimitrova N, Jakab Z, Kaatsch P, Lacour B, Mallone S, Marcos-Gragera R, Minicozzi P, Sanchez-Perez MJ, Sant M, Santaquilani M, Stiller C, Tavilla A, Trama A, Visser O, Peris-Bonet R, and the EUROCARE Working Group (2014) Childhood cancer survival in Europe 1999-2007: results of EUROCARE-5-a population-based study. The Lancet Oncology 15(1): 35-47

Gatta G, Capocaccia R, De Angelis R, Stiller C, Coebergh JW, and the EUROCARE Working Group (2003) Cancer survival in European adolescents and young adults. European journal of cancer (Oxford, England : 1990) 39(18): 2600-2610

Gatta G, Capocaccia R, Stiller C, Kaatsch P, Berrino F, Terenziani M, EUROCARE Working Group (2005) Childhood cancer survival trends in Europe: a EUROCARE Working Group Study. J Clin Oncol 23(16): 3742-3751

Gatta G, Corazziari I, Magnani C, Peris-Bonet R, Roazzi P, Stiller C, and the EUROCARE Working Group (2003) Childhood cancer survival in Europe. Annals of oncology 14(Suppl 5): v119-v127

Gatta G, Ferrari A, Stiller CA, Pastore G, Bisogno G, Trama A, Capocaccia R, The RARECARE Working Group (2012) Embryonal cancers in Europe. European journal of cancer (Oxford, England : 1990) 48(10): 1425-1433

Gatta G, Peris-Bonet R, Visser O, Stiller C, Marcos-Gragera R, Sánchez MJ, Lacour B, Kaatsch P, Berrino F, Rutkowski S, Botta L (2017) Geographical variability in survival of European children with central nervous system tumours. European Journal of Cancer 82: 137-148

Gatta G, Rossi S, Foschi R, Trama A, Marcos-Gragera R, Pastore G, Peris-Bonet R, Stiller C, Capocaccia R, and the EUROCARE Working Group (2013) Survival and cure trends for European children, adolescents and young adults diagnosed with acute lymphoblastic leukemia from 1982 to 2002. Haematologica 98(5): 744-752

Gatta G, Zigon G, Capocaccia R, Coebergh JW, Desandes E, Kaatsch P, Pastore G, Peris-Bonet R, Stiller CA, the Eurocare Working Group (2009) Survival of European children and young adults with cancer diagnosed 1995-2002. European journal of cancer (Oxford, England : 1990) 45(6): 992-1005

Gerrard M, Eden OB, Stiller CA (1986) Variations in incidence of childhood leukaemia in South East Scotland (1970-1984). Leuk Res 10(5): 561-564

Gibson BES, Eden OB, Barrett A, Stiller CA, Draper GJ (1988) Leukaemia in young children in Scotland. Lancet 332(8611): 630

Guérin S, Dupuy A, Anderson H, Shamsaldin A, Svahn-Tapper G, Moller T, Quiniou E, Garwicz S, Hawkins M, Avril MF, Oberlin O, Chavaudra J, de Vathaire F (2003) Radiation dose as a risk factor for malignant melanoma following childhood cancer. European journal of cancer (Oxford, England : 1990) 39(16): 2379-2386

Hanks S, Perdeaux ER, Seal S, Ruark E, Mahamdallie SS, Murray A, Ramsay E, Del Vecchio Duarte S, Zachariou A, de Souza B, Warren-Perry M, Elliott A, Davidson A, Price H, Stiller C, Pritchard-Jones K, Rahman N (2014) Germline mutations in the PAF1 complex gene CTR9 predispose to Wilms tumour. Nature communications 5: 4398

Hartley AL, Birch JM, McKinney PA, Blair V, Teare MD, Carrette J, Mann JR, Stiller CA, Draper GJ, Johnston HE, Cartwright RA, Waterhouse JAH (1988) The Inter-Regional Epidemiological Study of Childhood Cancer (IRESCC): past medical history in children with cancer. J Epidemiol Community Health 42(3): 235-242

Hartley AL, Birch JM, McKinney PA, Teare MD, Blair V, Carrette J, Mann JR, Draper GJ, Stiller CA, Johnston HE, Cartwright RA, Waterhouse JAH (1988) The Inter-Regional Epidemiological Study of Childhood Cancer (IRESCC): case control study of children with bone and soft tissue sarcomas. Br J Cancer 58(6): 838-842

Hasle H, Passmore SJ (2003) Epidemiology of MDS and Myeloproliferative Disorders in Children. In Myelodysplastic and Myeloproliferative Disorders in Children, Lopes LF, Hasle H (eds), Chapter 3, pp 49-66. Brazil: Tecmedd

Hawkins MM (1986) Second primary tumours among survivors of childhood cancer treated with anticancer drugs. IARC Sci Publ 78: 231-252

Hawkins MM (1989) Long term survival and cure after childhood cancer. Archives of disease in childhood 64(6): 798-807

Hawkins MM (1990) Risks of myeloid leukaemia in children treated for solid tumours. Lancet 336(8719): 887

Hawkins MM (1990) Second primary tumors following radiotherapy for childhood cancer. Int J Radiat Oncol Biol Phys 19(5): 1297-1301

Hawkins MM (1991) Is there evidence of a therapy-related increase in germ cell mutation among childhood cancer survivors? J Natl Cancer Inst 83(22): 1643-1650

Hawkins MM (1991) Secondary leukaemia after epipodophyllotoxins. Lancet 338(8779): 1408

Hawkins MM (1994) Pregnancy outcome and offspring after childhood cancer:  Data show no evidence of mutagenesis of the germ cells. BMJ 309(6961): 1034

Hawkins MM (1996) Multiple primary cancers in population-based cancer registries. European journal of cancer (Oxford, England : 1990) 32A(8): 1279-1280

Hawkins MM, Craft AW (1994) Retaining personal medical records of children who have had chemotherapy and radiotherapy. BMJ 308(6945): 1654-1655

Hawkins MM, Craft AW (1995) Use of personal records for research purposes. Records prematurely destroyed. BMJ 310(6974): 258

Hawkins MM, Draper GJ, Kingston JE (1987) Incidence of second primary tumours among childhood cancer survivors. Br J Cancer 56(3): 339-347

Hawkins MM, Draper GJ, Smith RA (1989) Cancer among 1,348 offspring of survivors of childhood cancer. Int J Cancer 43(6): 975-978

Hawkins MM, Draper GJ, Winter DL (1995) Cancer in the offspring of survivors of childhood leukaemia and non-Hodgkin lymphomas. Br J Cancer 71(6): 1335-1339

Hawkins MM, Draper GJ, Winter DL (1996) Heritability of childhood leukaemia and non-Hodgkin lymphoma. Br J Cancer 73(6): 848

Hawkins MM, Kingston JE (1988) Malignant thyroid tumours following childhood cancer. Lancet 332(8614): 804

Hawkins MM, Kingston JE, Kinnier Wilson LM (1989) Excess naevi after chemotherapy in childhood. BMJ 299(6695): 390

Hawkins MM, Kingston JE, Kinnier Wilson LM (1990) Late deaths after treatment for childhood cancer. Archives of disease in childhood 65(12): 1356-1363

Hawkins MM, Kinnier Wilson LM, Burton HS, Potok MHN, Winter DL, Marsden HB, Stovall MA (1996) Radiotherapy, alkylating agents, and risk of bone cancer after childhood cancer. J Natl Cancer Inst 88(5): 270-278

Hawkins MM, Kinnier Wilson LM, Stovall MA, Marsden HB, Potok MHN, Kingston JE, Chessells JM (1992) Epipodophyllotoxins, alkylating agents, and radiation and risk of secondary leukaemia after childhood cancer. BMJ 304(6832): 951-958

Hawkins MM, Robertson CM, Edge JA, Neil HAW (1996) Is risk of diabetes mellitus increased after abdominal radiotherapy? Lancet 347(9000): 539-540

Hawkins MM, Smith RA (1989) Pregnancy outcomes in childhood cancer survivors: probable effects of abdominal irradiation. Int J Cancer 43(3): 399-402

Hawkins MM, Smith RA, Curtice LJ (1988) Childhood cancer survivors and their offspring studied through a postal survey of general practitioners: preliminary results. J R Coll Gen Pract 38(308): 102-105

Hawkins MM, Stevens MCG (1996) The long-term survivors. Br Med Bull 52(4): 898-923

Hawkins MM, Swerdlow AJ (1992) Completeness of cancer and death follow-up obtained through the National Health Service Central Register for England and Wales. Br J Cancer 66(2): 408-413

Hawkins MM, Winter DL, Burton HS, Potok MHN (1995) Heritability of Wilms' tumour. J Natl Cancer Inst 87(17): 1323-1324

Higgins CD, dos-Santos-Silva I, Stiller CA, Swerdlow AJ (2001) Season of birth and diagnosis of children with leukaemia: an analysis of over 15000 UK cases occurring from 1953-95. Br J Cancer 84(3): 406-412

Honjo S, Doran HE, Stiller CA, Ajiki W, Tsukuma H, Oshima A, Coleman MP (2003) Neuroblastoma trends in Osaka, Japan, and Great Britain 1970-1994, in relation to screening. Int J Cancer 103(4): 538-543

Hopton PA, McKinney PA, Cartwright RA, Mann JR, Birch JM, Hartley AL, Waterhouse JA, Johnston HE, Draper GJ, Stiller CA (1985) X-rays in pregnancy and the risk of childhood cancer. Lancet 326(8458): 773

Ilgren EB, Kinnier Wilson LM, Stiller CA (1985) Gliomas in neurofibromatosis: a series of 89 cases with evidence for enhanced malignancy in associated cerebellar astrocytomas. Pathol Annu 20(Pt 1): 331-358

Ilgren EB, Stiller CA (1986) Cerebellar astrocytomas: therapeutic management. Acta neurochirurgica 81(1-2): 11-26

Ilgren EB, Stiller CA (1987) Cerebellar astrocytomas.  Clinical characteristics and prognostic indices. J Neurooncol 4(3): 293-308

Ilgren EB, Stiller CA (1987) Cerebellar astrocytomas. Part I. Macroscopic and microscopic features. Clin Neuropathol 6(5): 185-200

Ilgren EB, Stiller CA (1987) Cerebellar astrocytomas. Part II. Pathologic features indicative of malignancy. Clin Neuropathol 6(5): 201-214

Ilgren EB, Stiller CA, Hughes JT, Silberman D, Steckel N, Kaye A (1984) Ependymomas: a clinical and pathologic study. Part I. Biologic features. Clin Neuropathol 3(3): 113-121

Ilgren EB, Stiller CA, Hughes JT, Silberman D, Steckel N, Kaye A (1984) Ependymomas: a clinical and pathologic study. Part II. Survival features. Clin Neuropathol 3(3): 122-127


Jenkinson HC, Hawkins MM, Stiller CA, Winter DL, Marsden HB, Stevens MCG (2004) Long-term population-based risks of second malignant neoplasms after childhood cancer in Britain. Br J Cancer 91(11): 1905-1910

Jenkinson HC, Winter DL, Marsden HB, Stovall MA, Stevens MCG, Stiller CA, Hawkins MM (2007) A study of soft tissue sarcomas after childhood cancer in Britain. Br J Cancer 97(5): 695-699

Johnston HE, Mann JR, Williams J, Waterhouse JAH, Birch JM, Cartwright RA, Draper GJ, Hartley AL, McKinney PA, Hopton PA, Stiller CA (1986) The Inter-Regional, Epidemiological Study of Childhood Cancer (IRESCC): case-control study in children with germ cell tumours. Carcinogenesis 7(5): 717-722


Jones EA, Stewart A, Stiller C, Douglas F, Bown N (2011) Wilms tumor incidence in children with 2q terminal deletions: A cohort study. Am J Med Genet Part A 155A(9): 2221-2223

Kardos G, Baumann I, Passmore SJ, Locatelli F, Hasle H, Schultz KR, Starý J, Schmitt-Graeff A, Fischer A, Harbott J, Chessells JM, Hann I, Fenu S, Rajnoldi AC, Kerndrup G, van Wering E, Rogge T, Nöllke P, Niemeyer CM (2003) Refractory anemia in childhood: a retrospective analysis of 67 patients with particular reference to monosomy 7. Blood 102(6): 1997-2003

Keegan TJ, Bunch KJ, Vincent TJ, King JC, O'Neill KA, Kendall GM, MacCarthy A, Fear NT, Murphy MF (2013) Case-control study of paternal occupation and social class with risk of childhood central nervous system tumours in Great Britain, 1962-2006. Br J Cancer 108(9): 1907-1914

Keegan TJ, Bunch KJ, Vincent TJ, King JC, O'Neill KA, Kendall GM, MacCarthy A, Fear NT, Murphy MFG (2012) Case-control study of paternal occupation and risk of childhood leukaemia in Great Britain, 1962-2006. Br J Cancer 107(9): 1652-1659

Kendall G, Muirhead C, Draper GJ (1993) Parental exposure to radiation and childhood cancer. Radiol Prot Bull 147: 7-13

Kendall G, Murphy M (2007) Natural environmental radiation and childhood cancer. Environmental Radon Newsletter Autumn(52): 1

Kendall GM (2010) Radon: health effects of environmental exposures. In Environmental Medicine, Ayres JG, Harrison RM, Nichols GL, Maynard RL (eds), Chapter 56, pp 599-609. London: Hodder Arnold

Kendall GM, Bithell JF, Bunch KJ, Draper GJ, Kroll ME, Murphy MFG, Stiller CA, J. VT (2016) Sellafield and Other Clusters of Childhood Cancer in the Vicinity of Nuclear Installations. Radiation Environment and Medicine 5(1): 31-39

Kendall GM, Bunch KJ (2011) Cancer in the offspring of radiation workers, RAD August 2011. 

Kendall GM, Bunch KJ, Miles JCHM, Vincent TJ, Little MP, Wakeford R, Meara JR, Murphy MFG (2013) Report of a record-based case-control study of natural background radiation and incidence of childhood cancer in Great Britain Chilton,Didcot: Health Protection Agency. 

Kendall GM, Fell TP (2011) Doses to the red bone marrow of young people and adults from radiation of natural origin. J Radiol Prot 31(3): 329-335

Kendall GM, Fell TP, Harrison JD (2009) Dose to red bone marrow of infants, children and adults from radiation of natural origin. J Radiol Prot 29(2): 123-138

*Kendall GM, Green BMR, Miles JCH, Dixon DW (2005) The development of the UK radon programme. J Radiol Prot 25(4): 475-492

Kendall GM, Hughes JS, Oatway WB, Jones AL (2006) Variations in radiation exposures of adults and children in the UK. J Radiol Prot 26(3): 257-276

Kendall GM, Little MP, Wakeford R (2011) Numbers and proportions of leukemias in young people and adults induced by radiation of natural origin. Leuk Res 35(8): 1039-1043

Kendall GM, Little MP, Wakeford R, Bunch KJ, Miles JCH, Vincent TJ, Meara JR, G MMF (2016) A brief review of case-control studies of natural background radiation and childhood cancer in Great Britain. Radiation Environment and Medicine 5(1): 27-30

Kendall GM, Little MP, Wakeford R, Bunch KJ, Miles JCH, Vincent TJ, Meara JR, Murphy MF (2013) A record-based case-control study of natural background radiation and the incidence of childhood leukaemia and other cancers in Great Britain during 1980-2006. Leukemia 27(1): 3-9

Kendall GM, Miles JCH, Rees D, Wakeford R, Bunch KJ, Vincent TJ, Little MP (2016) Variation with socioeconomic status of indoor radon levels in Great Britain: The less affluent have less radon. Journal of Environmental Radioactivity 164: 84-90

Kendall GM, Phipps AW (2007) Effective and organ doses from thoron decay products at different ages. J Radiol Prot 27(4): 427-435

Kendall GM, Smith TJ (2005) Doses from radon and its decay products to children. J Radiol Prot 25(3): 241-256

Kendall GM, Wakeford R, Athanson M, Vincent TJ, Carter EJ, McColl NP, Little MP (2016) Levels of naturally occurring gamma radiation measured in British homes and their prediction in particular  residences. Radiation and Environmental Biophysics 55(1): 103-124

Kendall GM, Wakeford R, Bunch KJ, Vincent TJ, Little MP (2015) Residential mobility and associated factors in relation to the assessment of exposure to naturally occurring radiation in studies of childhood cancer. J Radiol Prot 35: 835–868

Kheifets L, Ahlbom A, Crespi CM, Draper G, Hagihara J, Lowenthal RM, Mezei G, Oksuzyan S, Schüz J, Swanson J, Tittarelli A, Vinceti M, Wunsch Filho V (2010) Pooled analysis of recent studies on magnetic fields and childhood leukaemia. Br J Cancer 103(7): 1128-1135

Kheifets L, Ahlbom A, Crespi CM, Draper G, Hagihara J, Lowenthal RM, Mezei G, Oksuzyan S, Schüz J, Swanson J, Tittarelli A, Vinceti M, Wunsch Filho V (2011) Pooled analysis of recent studies on magnetic fields and childhood leukaemia (erratum). Br J Cancer 104(1): 228

Kheifets L, Ahlbom A, Crespi CM, Feychting M, Johansen C, Monroe J, Murphy MF, Oksuzyan S, Preston-Martin S, Roman E, Saito T, Savitz D, Schüz J, Simpson J, Swanson J, Tynes T, Verkasalo P, Mezei G (2010) A pooled analysis of extremely low-frequency magnetic fields and childhood brain tumors. Am J Epidemiol 172(7): 752-761

Kheifets L, Ahlbom A, Crespi CM, Feychting M, Johansen C, Monroe J, Murphy MF, Oksuzyan S, Preston-Martin S, Roman E, Saito T, Savitz D, Schüz J, Simpson J, Swanson J, Tynes T, Verkasalo P, Mezei G (2011) Author's reply to "Re: "A Pooled Analysis of Extremely Low-Frequency Magnetic Fields and Childhood Brain Tumors" (Milham,S)". Am J Epidemiol 173(3): 360-361

Kingston JE, Draper GJ, Mann JR (1982) Hepatoblastoma and polyposis coli. Lancet 319(8269): 457

Kingston JE, Hawkins MM, Draper GJ, Marsden HB, Kinnier Wilson LM (1987) Patterns of multiple primary tumours in patients treated for cancer during childhood. Br J Cancer 56(3): 331-338

Kingston JE, Herbert A, Draper GJ, Mann JR (1983) Association between hepatoblastoma and polyposis coli. Archives of disease in childhood 58(12): 959-962

Kingston JE, Malpas JS, Stiller CA, Pritchard J, McElwain TJ (1984) Autologous bone marrow transplantation contributes to haemopoietic recovery in children with solid tumours treated with high dose melphalan. Br J Haematol 58(4): 589-595

Kinlen LJ, Dickson M, Stiller CA (1995) Childhood leukaemia and non-Hodgkin's lymphoma near large rural construction sites, with a comparison with Sellafield nuclear site. BMJ 310(6982): 763-768

Kinlen LJ, Doll R (2004) Population mixing and childhood leukaemia: Fallon and other US clusters. Br J Cancer 91(1): 1-3

Kinlen LJ, Hudson CM, Stiller CA (1991) Contacts between adults as evidence for an infective origin of childhood leukaemia: an explanation for the excess near nuclear establishments in west Berkshire? Br J Cancer 64(3): 549-554

Kinlen LJ, Stiller CA (1993) Population mixing and excess of childhood leukaemia. BMJ 306(6882): 930


Kramárová E, Stiller CA (1996) The international classification of childhood cancer. International journal of cancer 68(6): 759-765

Kramárová E, Stiller CA, Ferlay J, Parkin DM, Draper GJ, Michaelis J, Neglia JP, Qureshi S (1996) The International Classification of Childhood Cancer. IARC Technical Report No29. Lyon: IARC

Kroll ME, Carpenter LM, Murphy MFG, Stiller CA (2012) Effects of changes in diagnosis and registration on time trends in recorded childhood cancer incidence in Great Britain. Br J Cancer 107(7): 1159-1162

Kroll ME, Draper GJ, Stiller CA, Murphy MFG (2006) Childhood leukemia incidence in Britain, 1974–2000: time trends and possible relation to influenza epidemics. J Natl Cancer Inst 98(6): 417-420

Kroll ME, Draper GJ, Stiller CA, Murphy MFG (2006) RESPONSE: Re: Childhood leukemia incidence in Britain, 1974-2000: time trends and possible relation to influenza epidemics. JNCI Cancer Spectrum 98(23): 1746-1747

Kroll ME, Murphy MFG, Carpenter LM, Stiller CA (2011) Childhood cancer registration in Britain: capture-recapture estimates of completeness of ascertainment. Br J Cancer 104(7): 1227-1233

Kroll ME, Passmore SJ, Stiller CA, Draper GJ, Bayne AM, Brownbill PA, Bunch KJ, Vincent TJ, Murphy MFG (2004) Childhood Cancer - UK. In CancerStats Monograph 2004, Toms JR (ed), Chapter 9, pp 63-72. Cancer Research UK

Kroll ME, Stiller CA, Murphy MFG (2012) Reply to: 'Childhood leukaemia and socioeconomic status in England and Wales 1976-2005: evidence of higher incidence in relatively affluent communities persists over time'. Br J Cancer 107(1): 219-220

Kroll ME, Stiller CA, Murphy MFG (2012) Reply: 'Childhood leukaemia and socioeconomic status'. Br J Cancer 107(1): 216

Kroll ME, Stiller CA, Murphy MFG, Carpenter LM (2011) Childhood leukaemia and socioeconomic status in England and Wales 1976-2005: evidence of higher incidence in relatively affluent communities persists over time. Br J Cancer 105(11): 1783-1787

Kroll ME, Stiller CA, Richards S, Mitchell C, Carpenter LM (2012) Evidence for under-diagnosis of childhood acute lymphoblastic leukaemia in poorer communities within Great Britain. Br J Cancer 106(9): 1556-1559

Kroll ME, Swanson J, Vincent TJ, Draper GJ (2010) Childhood cancer and magnetic fields from high-voltage power lines in England and Wales: a case-control study. Br J Cancer 103(7): 1122-1127

Kynaston JA, Malcolm AJ, Craft AW, Davies SM, Morris Jones PH, King DJ, Mitchell CD, Oakhill A, Stiller CA (1993) Chemotherapy in the management of infantile fibrosarcoma. Med Pediatr Oncol 21(7): 488-493

La Vecchia C, Draper GJ, Franceschi S (1984) Childhood nonovarian female genital tract cancers in Britain, 1962-1978. Descriptive epidemiology and long-term survival. Cancer 54(1): 188-192

La Vecchia C, Morris HB, Draper GJ (1983) Malignant ovarian tumours in childhood in Britain,1962-78. Br J Cancer 48(3): 363-374

Le Vu B, de Vathaire F, Shamsaldin A, Hawkins MM, Grimaud E, Hardiman C, Diallo I, Vassal G, Bessa E, Campbell S, Panis X, Daly-Schveitzer N, Lagrange J-L, Zucker J-M, Eschwège F, Chavaudra J, Lemerle J (1998) Radiation dose, chemotherapy and risk of osteosarcoma after solid tumours during childhood. Int J Cancer 77(3): 370-377

Lennox EL, Draper GJ, Sanders BM (1975) Retinoblastoma: a study of natural history and prognosis of 268 cases. Br Med J 3(5986): 731-734

Lennox EL, Stiller CA, Morris Jones PH, Kinnier Wilson LM (1979) Nephroblastoma: treatment during 1970-3 and the effect on survival of inclusion in the first MRC trial. Br Med J 2(6190): 567-569

Levitt G, Anazodo A, Burch M, Bunch K (2009) Cardiac or cardiopulmonary transplantation in childhood cancer survivors: an increasing need? European journal of cancer (Oxford, England : 1990) 45(17): 3027-3034

Levitt G, Bunch KJ, Rogers CA, Whitehead B (1996) Cardiac transplantation in childhood cancer survivors in Great Britain. European journal of cancer (Oxford, England : 1990) 32A(5): 826-830

Levitt GA, Stiller CA, Chessells JM (1990) Prognosis of Down's syndrome with acute leukaemia. Archives of disease in childhood 65(2): 212-216

Lightfoot T, Bunch K, Ansell P, Murphy M (2005) Ovulation induction, assisted conception and childhood cancer. European journal of cancer (Oxford, England : 1990) 41(5): 715-724

Little MP, de Vathaire F, Charles MW, Hawkins MM, Muirhead CR (1997) Variations with time and age in the relative risks of solid cancer incidence after radiation exposure. J Radiol Prot 17(3): 159-177

Little MP, Hawkins MM, Charles MW, Hildreth NG (1992) Fitting the Armitage-Doll model to radiation-exposed cohorts and implications for population cancer risks. Radiat Res 132(2): 207-221

Little MP, Hawkins MM, Charles MW, Hildreth NG (1994) Corrections to the paper 'Fitting the Armitage-Doll model to radiation-exposed cohorts and implications for population cancer risks'. Radiat Res 137(1): 124-128

Little MP, Hawkins MM, Shore RE, Charles MW, Hildreth NG (1991) Time variations in the risk of cancer following irradiation in childhood. Radiat Res 126(3): 304-316

Little MP, Kleinerman RA, Stiller CA, Li G, Kroll ME, Murphy MFG (2012) Analysis of retinoblastoma age incidence data using a fully stochastic cancer model. Int J Cancer 130(3): 631-640

Little MP, Muirhead CR, Stiller CA (1996) Modelling lymphocytic leukaemia incidence in England and Wales using generalizations of the two-mutation model of carcinogenesis of Moolgavkar, Venzon and Knudson. Statistics in medicine 15(10): 1003-1022

Little MP, Muirhead CR, Stiller CA (1997) Modelling acute lymphocytic leukaemia using generalizations of the MVK two-mutation model of carcinogenesis: implied mutation rates and the likely role of ionising radiation. In Microdosimetry: An Interdisciplinary approach, Goodhead DT, O'Neill P, Menzel HG (eds), pp 244-247. 

Little MP, Wakeford R, Kendall GM (2009) Updated estimates of the proportion of childhood leukaemia incidence in Great Britain that may be caused by natural background ionising radiation. J Radiol Prot 29(4): 467-482

Little MP, Wakeford R, Kendall GM (2010) Reply to "Comment on 'Updated estimates of the proportion of childhood leukaemia incidence in Great Britain that may be caused by natural background ionising radiation'". J Radiol Prot 30(1): 99-101

Little MP, Wakeford R, Lubin JH, Kendall GM (2010) The statistical power of epidemiological studies analyzing the relationship between exposure to ionizing radiation and cancer, with special reference to childhood leukemia and natural background radiation. Radiat Res 174(3): 387-402

Lycett D, Munafò MR, Johnstone E, Murphy MFG, Aveyard P (2011) Weight change over eight years in relation to alcohol consumption in a cohort of continuing smokers and quitters. Nicotine and tobacco research 13(11): 1149-1154

Lyons RA, Monaghan SP, Heaven M, Littlepage BNC, Vincent TJ, Draper GJ (1995) Incidence of leukaemia and lymphoma in young people in the vicinity of the petrochemical plant at Baglan Bay, South Wales, 1974 to 1991. Occup Environ Med 52(4): 225-228

MacCarthy A, Bayne AM, Brownbill PA, Bunch KJ, Diggens NL, Draper GJ, Hawkins MM, Jenkinson HC, Kingston JE, Stiller CA, Vincent TJ, Murphy MF (2013) Second and subsequent tumours among 1927 retinoblastoma patients diagnosed in Britain 1951-2004. Br J Cancer 108(12): 2455-2463

MacCarthy A, Bayne AM, Draper GJ, Eatock EM, Kroll ME, Stiller CA, Vincent TJ, Hawkins MM, Jenkinson HC, Kingston JE, Neale R, Murphy MFG (2009) Non-ocular tumours following retinoblastoma in Great Britain 1951 to 2004. Br J Ophthalmol 93(9): 1159-1162

MacCarthy A, Birch JM, Draper GJ, Hungerford JL, Kingston JE, Kroll ME, Onadim Z, Stiller CA, Vincent TJ, Murphy MFG (2009) Retinoblastoma in Great Britain 1963-2002. Br J Ophthalmol 93(1): 33-37

MacCarthy A, Birch JM, Draper GJ, Hungerford JL, Kingston JE, Kroll ME, Stiller CA, Vincent TJ, Murphy MFG (2009) Retinoblastoma: treatment and survival in Great Britain 1963 to 2002. Br J Ophthalmol 93(1): 38-39

MacCarthy A, Bunch KJ, Fear NT, King JC, Vincent TJ, Murphy MFG (2009) Paternal occupation and retinoblastoma:  A case-control study based on data for Great Britain 1962-1999. Occup Environ Med 66(10): 644-649

MacCarthy A, Bunch KJ, Fear NT, King JC, Vincent TJ, Murphy MFG (2010) Paternal occupation and neuroblastoma: a case-control study based on cancer registry data for Great Britain 1962-1999. Br J Cancer 102(3): 615-619

MacCarthy A, Draper GJ, Steliarova-Foucher E, Kingston JE (2006) Retinoblastoma incidence and survival in European children (1978–1997). Report from the Automated Childhood Cancer Information System project. European journal of cancer (Oxford, England : 1990) 42(13): 2092-2102

Magnani C, Gatta G, Corazziari I, Kramarova E, Pastore G, Viscomi S, Stiller C (2001) Childhood malignancies in the EUROCARE study: the database and the methods of survival analysis. European journal of cancer (Oxford, England : 1990) 37(6): 678-686

Mahamdallie SS, Hanks S, Karlin KL, Zachariou A, Perdeaux ER, Ruark E, Shaw CA, Renwick A, Ramsay E, Yost S, Elliott A, Birch J, Capra M, Gray J, Hale J, Kingston J, Levitt G, McLean T, Sheridan E, Renwick A, Seal S, Stiller C, Sebire N, Westbrook TF, Rahman N (2015) Mutations in the transcriptional repressor REST predispose to Wilms tumor. Nature genetics 47(12): 1471-4

Mann JR, Dodd HE, Draper GJ, Waterhouse JAH, Birch JM, Cartwright RA, Hartley AL, McKinney PA, Stiller CA (1993) Congenital abnormalities in children with cancer and their relatives: results from a case-control study (IRESCC). Br J Cancer 68(2): 357-363

Mann JR, Stiller CA (1994) Changing pattern of incidence and survival in children with germ cell tumours (GCTs). Advances in the biosciences 91: 59-64

Marsden HB, Lennox EL, Lawler W, Kinnier-Wilson LM (1980) Bone metastases in childhood renal tumours. Br J Cancer 41(6): 875-879

Mathew RK, O'Kane R, Parslow R, Stiller C, Kenny T, Picton S, Chumas PD (2014) Comparison of survival between the UK and US after surgery for most common pediatric CNS tumors. Neuro-oncology 16(8): 1137-45

McKinney PA, Cartwright RA, Saiu JMT, Mann JR, Stiller CA, Draper GJ, Hartley AL, Hopton PA, Birch JM, Waterhouse JAH, Johnston HE (1987) The Inter-Regional Epidemiological Study of Childhood Cancer (IRESCC): a case control study of aetiological factors in leukaemia and lymphoma. Archives of disease in childhood 62(3): 279-287

McKinney PA, Cartwright RA, Stiller CA, Hopton PA, Mann JR, Birch JM, Hartley AL, Waterhouse JA, Johnston HE (1985) Inter-Regional Epidemiological Study of Childhood Cancer (IRESCC): childhood cancer and the consumption of Debendox and related drugs in pregnancy. Br J Cancer 52(6): 923-929

McKinney PA, Stiller CA (1986) Maternal smoking during pregnancy and the risk of childhood cancer. Lancet 328(8505): 519-520

McNally R, Alexander F, Bithell J, Draper G, Vincent T, Murphy M (2007) Space-time clustering of childhood cancer around the residence at birth.  Abstract O.074. Pediatr Blood Cancer 49(Suppl 6): 418-419

McNally R, Bithell J, Vincent T, Murphy M (2012) Cross-space-time clustering of childhood cancer in Great Britain: evidence for a common aetiology.  Abstract PP007:. Pediatr Blood Cancer 59(6): 1095

McNally RJ, Bunch KJ, Craft AW, Murphy MF (2015) Response to: Comment on 'Updated investigations of cancer excesses in individuals born or resident in the vicinity of Sellafield and Dounreay'. Br J Cancer 112(11): 1837

McNally RJQ, Alexander FE, Bithell JF (2006) Space-time clustering of childhood cancer in Great Britain: a national study, 1969-1993. Int J Cancer 118(11): 2840-2846

McNally RJQ, Alexander FE, Vincent TJ, Murphy MFG (2009) Spatial clustering of childhood cancer in Great Britain during the period 1969-1993. Int J Cancer 124(4): 932-936

McNally RJQ, Bithell JF, Vincent TJ, Murphy MFG (2009) Space-time clustering of childhood cancer around the residence at birth. Int J Cancer 124(2): 449-455

McNally RJQ, Blakey K, Parslow RC, James PW, Goméz Pozo B, Stiller C, Vincent TJ, Norman P, McKinney PA, Murphy MF, Craft AW, Feltbower RG (2012) Small-area analyses of bone cancer diagnosed in Great Britain provide clues to aetiology. BMC cancer 12: 270

McNally RJQ, Stiller C, Vincent TJ, Murphy MFG (2014) Cross-space-time clustering of childhood cancer in Great Britain: Evidence for a common aetiology. International Journal of Cancer Journal International du Cancer 134(1): 136-143

McWhirter WR, Stiller CA, Lennox EL (1989) Carcinomas in childhood. A registry-based study of incidence and survival. Cancer 63(11): 2242-2246

Menu-Branthomme A, Rubino C, Shamsaldin A, Hawkins MM, Grimaud E, Dondon M-G, Hardiman C, Vassal G, Campbell S, Panis X, Daly-Schveitzer N, Lagrange J-L, Zucker J-M, Chavaudra J, Hartman O, de Vathaire F (2004) Radiation dose, chemotherapy and risk of soft tissue sarcoma after solid tumours during childhood. Int J Cancer 110(1): 87-93

Metayer C, Milne E, Clavel J, Infante-Rivard C, Petridou E, Taylor M, Schüz J, Spector LG, Dockerty JD, Magnani C, Pombo-de-Oliveira MS, Sinnett D, Murphy M, Roman E, Monge P, Ezzat S, Mueller BA, Scheurer ME, Armstrong BK, Birch J, Kaatsch P, Koifman S, Lightfoot T, Bhatti P, Bondy ML, Rudant J, O'Neill K, Miligi L, Dessypris N, Kang AY, Buffler PA (2013) The Childhood Leukemia International Consortium. Cancer epidemiology 37(3): 336-347

Morris HB, La Vecchia C, Draper GJ (1984) Malignant epithelial tumors of the ovary in childhood: a clinicopathological study of 13 cases in Great Britain 1962-1978. Gynecol Oncol 19(3): 290-297

Morris HHB, La Vecchia C, Draper GJ (1985) Endodermal sinus tumor and embryonal carcinoma of the ovary in children. Gynecol Oncol 21(1): 7-17

Morris JA, Cowell JK, Stiller CA, Barratt A (1993) Retinoblastoma: a possible link with low level radiation. J Med Genet 30(5): 440-442

Mott MG, Mann JR, Stiller CA (1997) The United Kingdom Children's Cancer Study Group - the first 20 years of growth and development. European journal of cancer (Oxford, England : 1990) 33(9): 1448-1452

Muirhead CR, Bunch KJ, Hunter N, Draper GJ, Kendall GM, O'Hagan JA, Phillipson MA, Vincent TJ, Zhang W (2009) Cancer in the Offspring of Female Radiation Workers - a Record Linkage Study Chilton,Didcot: Health Protection Agency. 

Muirhead CR, Butland BK, Green BMR, Draper GJ (1991) Childhood leukaemia and natural radiation. Lancet 337(8739): 503-504

Muirhead CR, Butland BK, Green BMR, Draper GJ (1992) An analysis of childhood leukaemia and natural radiation in Britain. Radiat Prot Dosimetry 45(1/4): 657-660

Murphy MFG, Bithell JF, Stiller CA, Kendall GM, O'Neill KA (2013) Childhood and adult cancers: contrasts and commonalities. Maturitas 76(1): 95-98

Murphy MFG, Bunch KJ, Chen B, Hemminki K (2008) Reduced occurrence of childhood cancer in twins compared to singletons: protection but by what mechanism? Pediatr Blood Cancer 51(1): 62-65

Murphy MFG, Whiteman D, Hey K, Griffith M, Gill L, Goldacre MJ, Vincent TJ, Bunch KJ (2001) Childhood cancer incidence in a cohort of twin babies. Br J Cancer 84(11): 1460-1462

Narod SA, Hawkins MM, Robertson CM, Stiller CA (1997) Congenital anomalies and childhood cancer in Great Britain. Am J Hum Genet 60(3): 474-485

Narod SA, Stiller C, Lenoir GM (1991) An estimate of the heritable fraction of childhood cancer. Br J Cancer 63(6): 993-999

Neale RE, Mineau G, Whiteman DC, Brownbill PA, Murphy MFG (2005) Childhood and adult cancer in twins: evidence from the Utah genealogy. Cancer epidemiology, biomarkers & prevention 14(5): 1236-1240

Neale RE, Stiller CA, Bunch KJ, Mineau G, Murphy MFG (2013) Familial aggregation of childhood and adult cancer in the Utah genealogy. Int J Cancer 133(12): 2953-60

O'Hara C et al. (2015) Trends in survival for teenagers and young adults with cancer in the UK 1992-2006 European journal of cancer (Oxford, England : 1990) 51:2039-2048 doi:10.1016/j.ejca.2015.06.112

O'Neill KA, Murphy MFG, Bunch KJ, Puumala SE, Carozza SE, Chow EJ, Mueller BA, McLaughlin CC, Reynolds P, Vincent TJ, Von Behren J, Spector LG (2015) Infant birthweight and risk of childhood cancer: international population-based case control studies of 40 000 cases. International Journal of Epidemiology 44(1): 153-168

O'Kane R, Mathew R, Kenny T, Stiller C, Chumas P (2013) United Kingdom 30-day mortality rates after surgery for pediatric central nervous system tumors. J Neurosurg Pediatr 12(3): 227-234

O'Neill KA, Bunch KJ, Murphy MFG (2012) Intrauterine growth and childhood leukemia and lymphoma risk. Expert Rev Hematol 5(5): 559-576

O'Neill KA, Bunch KJ, Vincent TJ, Spector LG, Moorman AV, Murphy MFG (2012) Immunophenotype and cytogenetic characteristics in the relationship between birth weight and childhood leukemia. Pediatr Blood Cancer 58(1): 7-11

Owen PJ, Miles DPB, Draper GJ, Vincent TJ (2002) Retrospective study of mortality after a water pollution incident at Lowermoor in north Cornwall. BMJ 324(7347): 1189

Parkin DM, Cardis E, Masuyer E, Friedl HP, Hansluwka H, Bobev D, Ivanov E, Sinnaeve J, Augustin J, Plesko I, Storm HH, Rahu M, Karjalainen S, Bernard JL, Carli PM, L'Huillier MC, Lutz JM, Schaffer P, Schraub S, Michaelis J, Möhner M, Staneczek W, Vargha M, Crosignani P, Magnani C, Terracini B, Kriauciunas R, Coebergh JW, Langmark F, Zatonski W, Merabishvili V, Pompe-Kirn V, Barlow L, Raymond L, Black R, Stiller CA, Bennett BG (1993) Childhood leukaemia following the Chernobyl accident: the European Childhood Leukaemia-Lymphoma Incidence Study (ECLIS). European journal of cancer (Oxford, England : 1990) 29A(1): 87-95

Parkin DM, Clayton D, Black RJ, Masuyer E, Friedl HP, Ivanov E, Sinnaeve J, Tzvetansky CG, Geryk E, Storm HH, Rahu M, Pukkala E, Bernard JL, Carli PM, L'Huillier MC, Ménégoz F, Schaffer P, Schraub S, Kaatsch P, Michaelis J, Apjok E, Schüler D, Crosignani P, Magnani C, Terracini B, Stengrevics A, Kriauciunas R, Coebergh JW, Langmark F, Zatonski W, Tulbure R, Boukhny A, Merabishvili V, Plesko I, Kramárová E, Pompe-Kirn V, Barlow L, Enderlin F, Levi F, Raymond L, Schüler G, Torhorst J, Stiller CA, Sharp L, Bennett BG (1996) Childhood leukaemia in Europe after Chernobyl:  5 year follow-up. Br J Cancer 73(8): 1006-1012

Parkin DM, Kramárová E, Draper GJ, Masuyer E, Michaelis J, Neglia J, Qureshi S, Stiller CA, Kramárová E, Draper GJ (1998) International Incidence of Childhood Cancer, Volume 2. IARC Scientific Publications, No 144, IARC Scientific Publications, No 144 edn. Lyon: International Agency for Research on Cancer

Parkin DM, Stiller CA (1995) Childhood cancer in developing countries: environmental factors. Int J Pediatr Hematol/Oncol 2: 411-417

Parkin DM, Stiller CA, Draper GJ, Bieber CA (1988) The international incidence of childhood cancer. Int J Cancer 42(4): 511-520

Parkin DM, Stiller CA, Draper GJ, Bieber CA, Terracini B, Young JL (1988) International Incidence of Childhood Cancer. IARC Scientific Publications, No. 87, IARC Scientific Publications, No. 87 edn. Lyon: International Agency for Research on Cancer

Parkin DM, Stiller CA, Nectoux J (1993) International variations in the incidence of childhood bone tumours. Int J Cancer 53(3): 371-376

Parkin M, Nectoux J, Stiller C, Draper G (1989) Incidence of cancer in children throughout the world. Pédiatrie 44(9): 725-736

Passmore SJ, Chessells JM, Kempski H, Hann IM, Brownbill PA, Stiller CA (2003) Paediatric myelodysplastic syndromes and juvenile myelomoncytic leukaemia in the UK: a population-based study of incidence and survival. Br J Haematol 121(5): 758-767

Passmore SJ, Draper GJ, Brownbill PA, Kroll ME (1998) Case-control studies of relation between childhood cancer and neonatal vitamin K administration. BMJ 316(7126): 178-184

Passmore SJ, Draper GJ, Brownbill PA, Kroll ME (1998) Ecological studies of relation between hospital policies on neonatal vitamin K administration and subsequent occurrence of childhood cancer. BMJ 316(7126): 184-189

Passmore SJ, Draper GJ, Stiller CA (1993) Vitamin K and childhood cancer. BMJ 307(6912): 1140

Passmore SJ, Hann IM, Stiller CA, Ramani P, Swansbury GJ, Gibbons B, Reeves BR, Chessells JM (1995) Pediatric myelodysplasia: a study of 68 children and a new prognostic scoring system. Blood 85(7): 1742-1750

Plesko I, Kramárová E, Stiller CA, Coebergh J-W, Santaquilani M, EUROCARE Working Group (2001) Survival of children with Wilms' tumour in Europe. European journal of cancer (Oxford, England : 1990) 37(6): 736-743

Powell JE, Esteve J, Mann JR, Parker L, Frappaz D, Michaelis J, Kerbl R, Mutz ID, Stiller CA, on behalf of SENSE (1998) Neuroblastoma in Europe: differences in the pattern of disease in the UK. Lancet 352(9129): 682-687

Powell JE, Stevens MCG, Stiller CA (1995) Clustering of childhood peritoneal mesothelioma in the Midlands, UK. Lancet 345(8941): 66-67

Pritchard J, Stiller CA, Lennox EL (1989) Overtreatment of children with Wilms' tumour outside paediatric oncology centres. BMJ 299(6703): 835-836

Pritchard-Jones K, Hawkins MM (1997) Biology of Wilms' tumour. Lancet 349(9053): 663-664

Pritchard-Jones K, Kaatsch P, Steliarova-Foucher E, Stiller CA, Coebergh JWW (2006) Cancer in children and adolescents in Europe: developments over 20 years and future challenges. European journal of cancer (Oxford, England : 1990) 42(13): 2183-2190

Pritchard-Jones K, Stiller CA (2007) What can we learn from geographical comparisons of childhood cancer survival? Br J Cancer 96(10): 1493-1497

Rao A, Hills RK, Stiller C, Gibson BE, De Graaf SSN, Hann IM, O'Marcaigh A, Wheatley K, Webb DKH (2006) Treatment for myeloid leukaemia of Down syndrome: population-based experience in the UK and results from the Medical Research Council AML 10 and AML 12 trials. Br J Haematol 132(5): 576-583

Reulen RC, Frobisher C, Winter DL, Kelly J, Lancashire ER, Stiller CA, Pritchard-Jones K, Jenkinson HC, Hawkins MM, for the British Childhood Cancer Survivor Study Steering Group (2011) Long-term risks of subsequent primary neoplasms among survivors of childhood cancer. JAMA 305(22): 2311-2319

Reulen RC, Taylor AJ, Winter DL, Stiller CA, Frobisher C, Lancashire ER, McClanahan FM, Sugden EM, Hawkins MM, on behalf of the British Childhood Cancer Survivor Study (2008) Long-term population-based risks of breast cancer after childhood cancer. Int J Cancer 123(9): 2156-2163

Reulen RC, Winter DL, Frobisher C, Lancashire ER, Stiller CA, Jenney ME, Skinner R, Stevens MC, Hawkins MM, for the British Childhood Cancer Survivor Study Steering Group (2010) Long-term cause-specific mortality among survivors of childhood cancer. JAMA 304(2): 172-179

Richardson S, Monfort C, Green M, Draper GJ, Muirhead C (1995) Spatial variation of natural radiation and childhood leukaemia incidence in Great Britain. Stat Med 14(21-22): 2487-2501

Robertson CM, Hawkins MM (1995) Childhood cancer and cystic fibrosis. J Natl Cancer Inst 87(19): 1486-1487

Robertson CM, Hawkins MM, Kingston JE (1994) Late deaths and survival after childhood cancer: implications for cure. BMJ 309(6948): 162-166

Robertson CM, Stiller CA, Kingston JE (1992) Causes of death in children diagnosed with non-Hodgkin's lymphoma between 1974 and 1985. Archives of disease in childhood 67(11): 1378-1383

Roman E, Fear NT, Ansell P, Bull D, Draper G, McKinney P, Michaelis J, Passmore SJ, von Kries R (2002) Vitamin K and childhood cancer: analysis of individual patient data from six case-control studies. Br J Cancer 86(1): 63-69

Saha V, Eden T, Stiller CA, Brown RJ, Mackinlay G (1993) An audit of the activities of the paediatric oncology unit in Edinburgh, 1982-1991. Scott Med J 38(3): 74-76

Sanders BM, Draper GJ (1979) Childhood cancer and drugs in pregnancy. Br Med J 1(6165): 717-718

Sanders BM, Draper GJ, Kingston JE (1988) Retinoblastoma in Great Britain 1969-80: incidence, treatment, and survival. Br J Ophthalmol 72(8): 576-583

Sanders BM, Jay M, Draper GJ, Roberts EM (1989) Non-ocular cancer in relatives of retinoblastoma patients. Br J Cancer 60(3): 358-365

Sanders BM, White GC, Draper GJ (1981) Occupations of fathers of children dying from neoplasms. J Epidemiol Community Health 35(4): 245-250

Sankila R, Martos Jiménez MC, Miljus D, Pritchard-Jones K, Steliarova-Foucher E, Stiller C (2006) Geographical comparison of cancer survival in European children (1988-1997): report from the Automated Childhood Cancer Information System project. European journal of cancer 42(13): 1972-1980

Satgé D, Moore SW, Stiller CA, Niggli FK, Pritchard-Jones K, Bown N, Bénard J, Plantaz D (2003) Abnormal constitutional karyotypes in patients with neuroblastoma: a report of four new cases and reviews of 47 others in the literature. Cancer genetics and cytogenetics 147(2): 89-98

Satgé D, Sasco AJ, Carlsen NLT, Rubie H, Stiller CA (1997) A negative association between Down's syndrome and neuroblastoma. Archives of disease in childhood 76(1): 80

Satgé D, Sasco AJ, Carlsen NLT, Stiller CA, Rubie H, Hero B, De Bernardi B, de Kraker J, Coze C, Kogner P, Langmark F, Hakvoort-Cammel FGAJ, Beck D, von der Weid N, Parkes S, Hartmann O, Lippens RJJ, Kamps WA, Sommelet D (1998) A lack of neuroblastoma in Down Syndrome: a study from 11 European countries. Cancer Res 58(3): 448-452

Satgé D, Stiller CA, Rutkowski S, von Bueren AO, Lacour B, Sommelet D, Nishi M, Massimino M, Garré ML, Moreno F, Hasle H, Jakab Z, Greenberg M, von der Weid N, Kuehni C, Zurriaga O, Vicente ML, Peris-Bonet R, Benesch M, Vekemans M, Sullivan SG, Rickert C (2013) A very rare cancer in Down syndrome: medulloblastoma. Epidemiological data from 13 countries. J Neurooncol 112(1): 107-114

Schoot RA, Slater O, Ronckers CM, Zwinderman AH, Balm AJ, Hartley B, van den Brekel MW, Gupta S, Saeed P, Gajdosova E, Pieters BR, Gaze MN, Mandeville HC, Fajardo RD, Chang YC, Gains JE, Strackee SD, Dunaway D, Abela C, Mason C, Smeele LE, Chisholm JC, Levitt GA, Kremer LC, Grootenhuis MA, Maurice-Stam H, Stiller CA, Hammond P, Caron HN, Merks JH (2015) Adverse events of local treatment in long-term head and neck rhabdomyosarcoma survivors after external beam radiotherapy or AMORE treatment. European journal of cancer (Oxford, England : 1990) 51(11): 1424-34

Scott RH, Douglas J, Baskcomb L, Huxter N, Barker K, Hanks S, Craft A, Gerrard M, Kohler JA, Levitt GA, Picton S, Pizer B, Ronghe MD, Williams D, Factors Associated with Childhood Tumours (FACT) Collaboration, Cook JA, Pujol P, Maher ER, Birch JM, Stiller CA, Pritchard-Jones K, Rahman N (2008) Constitutional 11p15 abnormalities, including heritable imprinting center mutations, cause nonsyndromic Wilms tumor. Nature genetics 40(11): 1329-1334

Scott RH, Murray A, Baskcomb L, Turnbull C, Loveday C, Al-Saadi R, Williams R, Breatnach F, Gerrard M, Hale J, Kohler J, Lapunzina P, Levitt GA, Picton S, Pizer B, Ronghe MD, Traunecker H, Williams D, Kelsey A, Vujanic GM, Sebire NJ, Grundy P, Stiller CA, Pritchard Jones K, Douglas J, Rahman N (2012) Stratification of Wilms tumor by genetic and epigenetic analysis. Oncotarget 3(3): 327-335

Scott RH, Stiller CA, Walker L, Rahman N (2006) Syndromes and constitutional chromosomal abnormalities associated with Wilms tumour. J Med Genet 43(9): 705-715

Shah A, Diggens N, Stiller C, Murphy D, Passmore SJ, Murphy MFG (2011) Place of death and hospital care for children who died of cancer in England, 1999-2006. European journal of cancer (Oxford, England : 1990) 47(14): 2175-2181

Shah A, Diggens NL, Stiller CA, Richards S, Stevens M, Murphy MFG (2014) Recruitment of childhood leukaemia patients to clinical trials in Great Britain during 1980-2007: variation by birth weight, congenital malformation, socioeconomic status and ethnicity. Arch Dis Child 99: 407-412

Shah A, Stiller C, Lancaster D, Vincent T, Coleman MP (2010) Leukaemia survival trends in children with Down's syndrome in Great Britain, 1971-2000: a population-based study. J Epidemiol Community Health 64(7): 604-609

Shah A, Stiller CA, Kenward MG, Vincent T, Eden TOB, Coleman MP (2008) Childhood leukaemia: long-term excess mortality and the proportion 'cured'. Br J Cancer 99(1): 219-223

Slade I, Bacchelli C, Davies H, Murray A, Abbaszadeh F, Hanks S, Barfoot R, Burke A, Chisholm J, Hewitt M, Jenkinson H, King D, Morland B, Pizer B, Prescott K, Sagger A, Side L, Traunecker H, Vaidya S, Ward P, Futreal PA, Vujanic G, Nicholson AG, Sebire N, Turnbull C, Priest JR, Pritchard-Jones K, Houlston R, Stiller C, Stratton MR, Douglas J, Rahman N (2011) DICER1 syndrome: clarifying the diagnosis, clinical features and management implications of a pleiotropic tumour predisposition syndrome. J Med Genet 48(4): 273-278

Slade I, Murray A, Hanks S, Kumar A, Walker L, Hargrave D, Douglas J, Stiller C, Izatt L, Rahman N (2011) Heterogeneity of familial medulloblastoma and contribution of germline PTCH1 and SUFU mutations to sporadic medulloblastoma. Fam Cancer 10(2): 337-342

Slade I, Stephens P, Douglas J, Barker K, Stebbings L, Abbaszadeh F, Pritchard-Jones K, FACT collaboration, Cole R, Pizer B, Stiller C, Vujanic G, Scott RH, Stratton MR, Rahman N (2010) Constitutional translocation breakpoint mapping by genome-wide paired-end sequencing identifies HACE1 as a putative Wilms tumor susceptibility gene. J Med Genet 47(5): 342-347

Smith RA, Hawkins MM (1989) Pregnancies after childhood cancer. Br J Obstet Gynaecol 96(4): 378-380

Sorahan T, Haylock RGE, Muirhead CR, Bunch KJ, Kinlen LJ, Little MP, Draper GJ, Kendall GM, Lancashire RJ, English MA (2003) Cancer in the offspring of radiation workers: an investigation of employment timing and a reanalysis using updated dose information. Br J Cancer 89(7): 1215-1220

Sorahan T, McKinney PA, Mann JR, Lancashire RJ, Stiller CA, Birch JM, Dodd HE, Cartwright RA (2001) Childhood cancer and parental use of tobacco:  findings from the inter-regional epidemiological study of childhood cancer (IRESCC). Br J Cancer 84(1): 141-146

Spix C, Aareleid T, Stiller C, Magnani C, Kaatsch P, Michaelis J (2001) Survival of children with neuroblastoma:  time trends and regional differences in Europe, 1978-1992. European journal of cancer (Oxford, England : 1990) 37(6): 722-729

Spix C, Pastore G, Sankila R, Stiller CA, Steliarova-Foucher E (2006) Neuroblastoma incidence and survival in European children (1978-1997): report from the Automated Childhood Cancer Information System project. European journal of cancer (Oxford, England : 1990) 42(13): 2081-2091

Spriggs AI, Draper GJ (1975) Calculation of positive rate from multiple samples. Acta Cytol 19(4): 319-320


Stark D, Bowen D, Dunwoodie E, Feltbower R, Johnson R, Moran A, Stiller C, O'Hara C (2015) Survival patterns in teenagers and young adults with cancer in the United Kingdom: Comparisons with younger and older age groups. European journal of cancer (Oxford, England : 1990) 51(17): 2643-54

Steliarova-Foucher E, Colombet M, Ries LAG, Moreno F, Dolya A, Bray F, Hesseling P, Shin HY, Stiller CA (2017) International incidence of childhood cancer, 2001-10: a population-based registry study. The Lancet Oncology 18(6): 719-731

Steliarova-Foucher E, Stiller C, Colombet M, Kaatsch P, Zanetti R, Peris-Bonet R (2015) Registration of childhood cancer: Moving towards pan-European coverage? European journal of cancer (Oxford, England : 1990) 51(9): 1064-79

Steliarova-Foucher E, Stiller C, Kaatsch P, Berrino F, Coebergh JW, on behalf of the ACCIS Scientific Committee (2005) Trends in childhood cancer incidence in Europe, 1970-99. Lancet 365(9477): 2088

Steliarova-Foucher E, Stiller C, Kaatsch P, Berrino F, Coebergh J-W, Lacour B, Parkin M (2004) Geographical patterns and time trends of cancer incidence and survival among children and adolescents in Europe since the 1970s (the ACCIS project): an epidemiological study. Lancet 364(9451): 2097-2105

Steliarova-Foucher E, Stiller C, Lacour B, Kaatsch P (2005) International Classification of Childhood Cancer, third edition. Cancer 103(7): 1457-1467

Steliarova-Foucher E, Stiller CA, Pukkala E, Lacour B, Plesko I, Parkin DM (2006) Thyroid cancer incidence and survival among European children and adolescents (1978-1997): report from the Automated Childhood Cancer Information System project. European journal of cancer (Oxford, England : 1990) 42(13): 2150-2169

Stiller C (2002) Epidemiology of cancer in adolescents. Med Pediatr Oncol 39(3): 149-155

Stiller C (2007) Childhood cancer in Britain: incidence, survival, mortality. Oxford: Oxford University Press

Stiller C, Shah A (2012) The epidemiology of cancer in children and adolescents. In Cancer in children.  Clinical management, Stevens MCG, Caron HN, Biondi A (eds), Sixth edn, Chapter 1, pp 1-13. Oxford: Oxford University Press

Stiller CA (1985) Descriptive epidemiology of childhood leukaemia and lymphoma in Great Britain. Leuk Res 9(6): 671-674

Stiller CA (1988) Centralisation of treatment and survival rates for cancer. Archives of disease in childhood 63(1): 23-30

Stiller CA (1988) Treatment of osteosarcoma. Lancet 331(8591): 931

Stiller CA (1989) Sudden infant death syndrome. BMJ 298(6678): 958

Stiller CA (1989) Survival of patients with cancer. BMJ 299(6707): 1058-1059

Stiller CA (1990) Treatment protocols, specialist centres, and end results. BMJ 300(6716): 48-49

Stiller CA (1992) Aetiology and epidemiology. In Paediatric Oncology Clinical practice and controversies, Plowman PN, Pinkerton CR (eds), pp 1-24. London: Chapman & Hall Medical

Stiller CA (1992) Survival of patients in clinical trials and at specialist centres. In New Treatments for Cancer: Practical, Ethical and Legal Problems, Williams CJ (ed), Chapter 9, pp 120-136. John Wiley & Sons Ltd

Stiller CA (1993) Cancer registration: its uses in research, and confidentiality in the EC. J Epidemiol Community Health 47(5): 342-344

Stiller CA (1993) Retinoblastoma and low level radiation.  Twenty times more common in children whose mothers have lived in Seascale. BMJ 307(6902): 461-462

Stiller CA (1993) Trends in neuroblastoma in Great Britain: incidence and mortality, 1971-1990. European journal of cancer (Oxford, England : 1990) 29A(7): 1008-1012

Stiller CA (1994) Cancer in adolescence.  Special problems; special solutions. BMJ 308(6941): 1382-1383

Stiller CA (1994) Centralised treatment, entry to trials and survival. Br J Cancer 70(2): 352-362

Stiller CA (1994) International variations in the incidence of childhood carcinomas. Cancer epidemiology, biomarkers & prevention 3(4): 305-310

Stiller CA (1994) Kaposi's sarcoma and volcanic soils. Lancet 343(8891): 231

Stiller CA (1994) Malignancies. In The Epidemiology of Childhood Disorders, Pless IB (ed), Chapter 16, pp 439-472. New York: Oxford University Press

Stiller CA (1994) Population based survival rates for childhood cancer in Britain, 1980-91. BMJ 309(6969): 1612-1616

Stiller CA (1995) Non-specialist units, clinical trials and survival from testicular cancer. European journal of cancer (Oxford, England : 1990) 31A(3): 289-291

Stiller CA (1997) Aetiology and Epidemiology. In Pediatric Oncology, Pinkerton CR, Plowman PN (eds), 2nd edn, Chapter 1, pp 3-26. London: Chapman and Hall

Stiller CA (1997) Reliability of cancer registration data. European journal of cancer (Oxford, England : 1990) 33(6): 812-814

Stiller CA (1998) What causes Hodgkin's disease in children? European journal of cancer (Oxford, England : 1990) 34(4): 523-528

Stiller CA (2001) Thyroid cancer following Chernobyl. European journal of cancer (Oxford, England : 1990) 37(8): 945-947

Stiller CA (2004) Aetiology and Epidemiology. In Paediatric Oncology, Pinkerton CR, Plowman PN, Pieters R (eds), 3rd Edition edn, Chapter 1, pp 3-24. London: Arnold

Stiller CA (2004) Epidemiology and genetics of childhood cancer. Oncogene 23(38): 6429-6444

Stiller CA (2005) Constitutional chromosomal abnormalities and childhood cancer. Italian journal of pediatrics 31(6): 347-353

Stiller CA (2005) Thyroid cancer in Belarus. Int J Epidemiol 34(3): 714

Stiller CA (2007) International patterns of cancer incidence in adolescents. Cancer treatment reviews 33(7): 631-645

Stiller CA (2008) Epidemiology of childhood tumours. In The Surgery of Childhood Tumours, Carachi R, Grosfeld JL, Azmy AF (eds). Heidelberg: Springer-Verlag

Stiller CA (2008) Pediatric cancers. In International Encyclopaedia of Public Health, Heggenhougen HK, Quah SR (eds) Vol. 5, pp 28-40. San Diego: Academic Press

Stiller CA, Allen MB, Brownbill PA, Draper GJ, Eatock EM, Loach MJ, Vincent TJ (1998) United Kingdom:  National Registry of Childhood Tumours, England and Wales, 1981-1990. In International Incidence of Childhood Cancer:  Volume 2, Parkin DM, Kramárová E, Draper GJ, Masuyer E, Michaelis J, Qureshi S, Stiller CA (eds), Chapter 4, pp 365-367. Lyon: IARC Scientific Publications No 144

Stiller CA, Allen MB, Eatock EM (1995) Childhood cancer in Britain: the National Registry of Childhood Tumours and incidence rates 1978-1987. European journal of cancer (Oxford, England : 1990) 31A(12): 2028-2034

Stiller CA, Benjamin S, Cartwright RA, Clough JV, Gorst DW, Kroll ME, Ross JRY, Wheatley K, Whittaker JA, Taylor PRA, Proctor SJ (1999) Patterns of care and survival for adolescents and young adults with acute leukaemia - a population based study. Br J Cancer 79(3/4): 658-665

Stiller CA, Bielack SS, Jundt G, Steliarova-Foucher E (2006) Bone tumours in European children and adolescents, 1978–1997. Report from the Automated Childhood Cancer Information System project. European journal of cancer (Oxford, England : 1990) 42(13): 2124-2135

Stiller CA, Bleyer WA (2004) Epidemiology. In Brain and Spinal Tumors of Childhood, Walker DA, Perilongo G, Punt JAG, Taylor RE (eds), Chapter 3, pp 35-49. Hodder Arnold

Stiller CA, Boyle PJ (1996) Effect of population mixing and socioeconomic status in England and Wales, 1979-85, on lymphoblastic leukaemia in children. BMJ 313(7068): 1297-1300

Stiller CA, Bunch KJ (1990) Trends in survival for childhood cancer in Britain diagnosed 1971-85. Br J Cancer 62(5): 806-815

Stiller CA, Bunch KJ (1992) Brain and spinal tumours in children aged under two years: incidence and survival in Britain, 1971-85. Br J Cancer 66(Suppl. XVIII): S50-S53

Stiller CA, Bunch KJ, Lewis IJ (2000) Ethnic group and survival from childhood cancer:  report from the UK Children's Cancer Study Group. Br J Cancer 82(7): 1339-1343

Stiller CA, Chessells JM, Fitchett M (1994) Neurofibromatosis and childhood leukaemia/lymphoma: a population-based UKCCSG study. Br J Cancer 70(5): 969-972

Stiller CA, Craft AW, Corazziari I, EUROCARE Working Group (2001) Survival of children with bone sarcoma in Europe since 1978: results from the EUROCARE study. European journal of cancer (Oxford, England : 1990) 37(6): 760-766

Stiller CA, Desandes E, Danon SE, Izarzugaza I, Ratiu A, Vassileva-Valerinova Z, Steliarova-Foucher E (2006) Cancer incidence and survival in European adolescents (1978–1997). Report from the Automated Childhood Cancer Information System project. European journal of cancer (Oxford, England : 1990) 42(13): 2006-2018

Stiller CA, Draper GJ (1982) Trends in childhood leukaemia in Britain 1968-1978. Br J Cancer 45(4): 543-551

Stiller CA, Draper GJ (1989) Treatment centre size, entry to trials, and survival in acute lymphoblastic leukaemia. Archives of disease in childhood 64(5): 657-661

Stiller CA, Draper GJ (2005) The epidemiology of cancer in children. In Cancer in children: clinical management, Voûte PA, Barrett A, Stevens MCG, Caron HN (eds), 5th edn, Chapter 1, pp 1-16. Oxford: Oxford University Press

Stiller CA, Draper GJ, Vincent TJ, O'Connor CM (1991) Incidence rates nationally and in administratively defined areas. In The Geographical Epidemiology of Childhood Leukaemia and Non-Hodgkin Lymphomas in Great Britain, 1966-83,OPCS Studies on Medical and Population Subjects No.53, Draper GJ (ed), Chapter 4, pp 25-35. London: HMSO

Stiller CA, Eatock EM (1994) Survival from acute non-lymphocytic leukaemia, 1971-88: a population based study. Archives of disease in childhood 70(3): 219-223

Stiller CA, Eatock EM (1999) Patterns of care and survival for children with acute lymphoblastic leukaemia diagnosed between 1980-94. Archives of disease in childhood 81(3): 202-208

Stiller CA, Kemp I, Draper GJ, Fearnley H, Lennox EL, Roberts EM, Sanders BM (1988) United Kingdom - Scotland.   National registry of childhood tumours, 1971-1980. In International incidence of childhood cancer, Parkin DM, Stiller CA, Draper GJ, Bieber CA, Terracini B, Young JL (eds), 87 edn, pp 305-308. Lyon: IARC Scientific Publications

Stiller CA, Kinnier Wilson LM (1981) Down syndrome and leukaemia. Lancet 318(8259): 1343

Stiller CA, Kroll ME, Boyle PJ, Feng Z (2008) Population mixing, socioeconomic status and incidence of childhood acute lymphoblastic leukaemia in England and Wales: analysis by census ward. Br J Cancer 98(5): 1006-1011

Stiller CA, Kroll ME, Boyle PJ, Feng Z (2008) Reply: Population change, population mixing and incidence of childhood acute lymphoblastic leukaemia in England and Wales. Br J Cancer 99(7): 1192-1193

Stiller CA, Kroll ME, Pritchard-Jones K (2012) Population survival from childhood cancer in Britain during 1978-2005 by eras of entry to clinical trials. Ann Oncol 23(9): 2464-2469

Stiller CA, Lennox EL (1980) Nephroblastoma in infants, 1969-75: variations in treatment and survival. Br Med J 281(6250): 1246-1248

Stiller CA, Lennox EL (1983) Childhood medulloblastoma in Britain 1971-77: analysis of treatment and survival. Br J Cancer 48(6): 835-841

Stiller CA, Lennox EL, Kinnier Wilson LM (1987) Incidence of cardiac septal defects in children with Wilms' tumour and other malignant diseases. Carcinogenesis 8(1): 129-132

Stiller CA, Marcos-Gragera R, Ardanaz E, Panelli F, Almar Marqués E, Cañada Martinez A, Steliarova-Foucher E (2006) Geographical patterns of childhood cancer incidence in Europe, 1988–1997. Report from the Automated Childhood Cancer Information System project. European journal of cancer 42(13): 1952-1960

Stiller CA, McKinney PA, Bunch KJ, Bailey CC, Lewis IJ (1991) Childhood cancer and ethnic group in Britain: a United Kingdom Children's Cancer Study Group (UKCCSG) study. Br J Cancer 64(3): 543-548

Stiller CA, Nectoux J (1994) International incidence of childhood brain and spinal tumours. Int J Epidemiol 23(3): 458-464

Stiller CA, O'Connor CM, Vincent TJ, Draper GJ (1991) The National Registry of Childhood Tumours and the leukaemia/lymphoma data for 1966-83. In The Geographical Epidemiology of Childhood Leukaemia and Non-Hodgkin Lymphomas in Great Britain, 1966-83,OPCS  Studies on Medical and Population Subjects No.53, Draper GJ (ed), Chapter 2, pp 7-16. London: HMSO

Stiller CA, Parkin DM (1990) International variations in incidence of childhood lymphomas. Paediatr Perinat Epidemiol 4(3): 303-324

Stiller CA, Parkin DM (1990) International variations in the incidence of childhood renal tumours. Br J Cancer 62(6): 1026-1030

Stiller CA, Parkin DM (1992) International variations in the incidence of neuroblastoma. Int J Cancer 52(4): 538-543

Stiller CA, Parkin DM (1994) International variations in the incidence of childhood soft-tissue sarcomas. Paediatr Perinat Epidemiol 8(1): 107-119

Stiller CA, Parkin DM (1996) Geographic and ethnic variations in the incidence of childhood cancer. Br Med Bull 52(4): 682-703

Stiller CA, Passmore SJ, Kroll ME, Brownbill PA, Wallis JC, Craft AW (2006) Patterns of care and survival for patients aged under 40 years with bone sarcoma in Britain, 1980-1994. Br J Cancer 94(1): 22-29

Stiller CA, Pritchard J, Steliarova-Foucher E (2006) Liver cancer in European children: incidence and survival, 1978-1997. Report from the Automated Childhood Cancer Information System project. European journal of cancer (Oxford, England : 1990) 42(13): 2115-2123

Stiller CA, Quinn M, Rowan S (2004) Childhood Cancer. In The health of children and young people, Chapter 13, pp 1-19. London: Office for National Statistics

Stiller CA, Stevens MCG, Magnani C, Corazziari I, EUROCARE Working Group (2001) Survival of children with soft-tissue sarcoma in Europe since 1978: results from the EUROCARE study. European journal of cancer (Oxford, England : 1990) 37(6): 767-774

Stiller CA, Trama A, Serraino D, Rossi S, Navarro C, Chirlaque MD, Casali PG, The RARECARE Working Group (2013) Descriptive Epidemiology of Sarcomas in Europe.  Report from the RARECARE Project. European journal of cancer (Oxford, England : 1990) 49(3): 684-695

Stiller CA, Olshan AF (2014) Epidemiology of renal tumors of childhood. In: Pritchard-Jones K, Dome JS (eds) Renal Tumors of Childhood: Biology and Therapy. Springer-Verlag, Heidelberg, 

Swanson J, Bunch KJ, Vincent TJ, Murphy MF (2014) Childhood cancer and exposure to corona ions from power lines: an epidemiological test. J Radiol Prot 34(4): 873-89

Swanson J, Bunch KJ, Vincent TJ, Murphy MF (2015) Reply to 'Comment on: Childhood cancer and exposure to corona ions from power lines: an epidemiological study'. J Radiol Prot 35(2): 485-6

Swanson J, Vincent T, J., Bunch K, J. (2014) Relative accuracy of grid references derived from postcode and address in UK epidem iological studies of overhead powerlines. J Radiol Prot 34: N81-N86

Swanson J, Vincent T, Kroll M, Draper G (2006) Power-frequency electric and magnetic fields in the light of Draper et al. 2005. Ann N Y Acad Sci 1076(1): 318-330

Swerdlow AJ, Stiller CA, Kinnier Wilson LM (1982) Prenatal factors in the aetiology of testicular cancer: an epidemiological study of childhood testicular cancer deaths in Great Britain, 1953-73. J Epidemiol Community Health 36(2): 96-101

Taylor AJ, Croft AP, Palace AM, Winter DL, Reulen RC, Stiller CA, Stevens MCG, Hawkins MM (2009) Risk of thyroid cancer in survivors of childhood cancer: Results from the British Childhood Cancer Survivor Study. Int J Cancer 125(10): 2400-2405

Taylor AJ, Frobisher C, Ellison DW, Reulen RC, Winter DL, Taylor RE, Stiller CA, Lancashire ER, Tudor EC, Baggott C, May S, Hawkins MM (2009) Survival after second primary neoplasms of the brain or spinal cord in survivors of childhood cancer:  Results from the British Childhood Cancer Survivor Study. J Clin Oncol 27(34): 5781-5787

Taylor AJ, Little MP, Winter DL, Sugden E, Ellison DW, Stiller CA, Stovall M, Frobisher C, Lancashire ER, Reulen RC, Hawkins MM (2010) Population-based risks of CNS tumors in survivors of childhood cancer: The British Childhood Cancer Survivor Study. J Clin Oncol 28(36): 5287-5293

Taylor AJ, Winter DL, Pritchard-Jones K, Stiller CA, Frobisher C, Lancashire ER, Reulen RC, Hawkins MM, on behalf of the British Childhood Cancer Survivor Study (2008) Second primary neoplasms in survivors of Wilms' tumour - a population-based cohort study from the British Childhood Cancer Survivor Study. Int J Cancer 122(9): 2085-2093

Taylor AJ, Winter DL, Stiller CA, Murphy MFG, Hawkins MM (2007) Risk of breast cancer in female survivors of childhood Hodgkin's disease in Britain: A population-based study. Int J Cancer 120(2): 384-391

Terracini B, Coebergh J-W, Gatta G, Magnani C, Stiller C, Verdecchia A, Zappone A (2001) Childhood cancer survival in Europe: an overview. European journal of cancer 37(6): 810-816

Toms JR, Draper GJ, Stiller CA, Adelstein AM, Donnan SPB, Fox AJ, MacDonald-Davies IM, White GC (1981) Cancer statistics:  Incidence, survival and mortality in England and Wales, OPCS Studies on Medical and Population Subjects No 43. London: HMSO

Trama A et al. (2016) Survival of European adolescents and young adults diagnosed with cancer in 2000-07: population-based data from EUROCARE-5 The Lancet Oncology 17:896-906 doi:10.1016/s1470-2045(16)00162-5

Turnbull C, Perdeaux ER, Pernet D, Naranjo A, Renwick A, Seal S, Munoz-Xicola RM, Hanks S, Slade I, Zachariou A, Warren-Perry M, Ruark E, Gerrard M, Hale J, Hewitt M, Kohler J, Lane S, Levitt G, Madi M, Morland B, Neefjes V, Nicholson J, Picton S, Pizer B, Ronghe M, Stevens M, Traunecker H, Stiller CA, Pritchard-Jones K, Dome J, Grundy P, Rahman N (2012) A genome-wide association study identifies susceptibility loci for Wilms tumor. Nature genetics 44(6): 681-684

Visser O, Trama A, Maynadié M, Stiller C, Marcos-Gragera R, De Angelis R, Mallone S, Tereanu C, Allemani C, Ricardi U, Schouten HC, The RARECARE Working Group (2012) Incidence, survival and prevalence of myeloid malignancies in Europe. European journal of cancer (Oxford, England : 1990) 48(17): 3257-3266

Wakeford R, Bithell JF (2015) Childhood cancer--the role of birthweight and antenatal radiography. Int J Epidemiol 44(5): 1741-3

Wakeford R, Darby SC, Murphy MFG (2010) Temporal trends in childhood leukaemia incidence following exposure to radioactive fallout from atmospheric nuclear weapons testing. Radiat Environ Biophys 49(2): 213-227

Wakeford R, Kendall GM, Little MP (2009) The proportion of childhood leukaemia incidence in Great Britain that may be caused by natural background ionizing radiation. Leukemia 23(4): 770-776

Wakeford R, Kendall GM, Little MP (2009) The risk of cancer from natural background ionizing radiation. Health Phys 97(6): 637-638

Wakeford R, Little MP, Kendall GM (2010) Risk of childhood leukemia after low-level exposure to ionizing radiation. Expert Rev Hematol 3(3): 251-254

Walker DA, Bendel A, Stiller C, Byrne P, Sokal M (2008) Central nervous system tumours. In Cancer in Adolescents and Young Adults, Bleyer A, Barr R (eds). Heidelberg: Springer-Verlag

Walker DA, Grundy R, Stiller C (2002) Rare Tumours of Childhood. In Oxford Textbook of Oncology Volume 2, Souhami RL, Tannock I, Hohenberger P, Horoit J-C (eds), 2nd edn, Chapter 17.7, pp 2669-2691. Oxford: Oxford University Press

Wall BF, Kendall GM, Edwards AA, Bouffler S, Muirhead CR, Meara JR (2006) What are the risks from medical X-rays and other low dose radiation? Br J Radiol 79(940): 285-294

Williams CL, Bunch KJ, Murphy MFG, Stiller CA, Botting BJ, Wallace WH, Davies MC, Sutcliffe AG (2017) Cancer risk in children born after donor ART. http://dx.doi.org/10.1093/humrep/dex333

Williams CL, Bunch KJ, Stiller CA, Murphy MFG, Botting BJ, Wallace H, Davies M, Sutcliffe AG (2013) Cancer risk among children born after assisted conception. The New England journal of medicine 369(19): 1819-1827

Williams CL, Bunch KJ, Sutcliffe AG (2014) Cancer risk among children born after assisted conception. The New England journal of medicine 370(10): 975-6

Windsor R, Stiller C, Webb D (2008) Peripheral T-Cell Lymphoma in Childhood: Population-Based Experience in the United Kingdom Over 20 Years. Pediatr Blood Cancer 50(4): 784-787


Wray NR, Alexander FE, Muirhead CR, Pukkala E, Schmidtmann I, Stiller CA (1999) A comparison of some simple methods to identify geographical areas with excess incidence of a rare disease such as childhood leukaemia. Statistics in medicine 18(12): 1501-1516
